# Effect of Heart Structure on Ventricular Fibrillation in the Rabbit: A Simulation Study

**DOI:** 10.3389/fphys.2019.00564

**Published:** 2019-05-15

**Authors:** Suran K. Galappaththige, Pras Pathmanathan, Martin J. Bishop, Richard A. Gray

**Affiliations:** ^1^Office of Science and Engineering Laboratories, Center for Devices and Radiological Health, U.S. Food and Drug Administration, Silver Spring, MD, United States; ^2^Division of Imaging Sciences, Department of Biomedical Engineering, King's College London, London, United Kingdom

**Keywords:** rabbit, model, cardiac arrhythmia, filament dynamics, fibrillation, phase mapping

## Abstract

Ventricular fibrillation (VF) is a lethal condition that affects millions worldwide. The mechanism underlying VF is unstable reentrant electrical waves rotating around lines called filaments. These complex spatio-temporal patterns can be studied using both experimental and numerical methods. Computer simulations provide unique insights including high resolution dynamics throughout the heart and systematic control of quantities such as fiber orientation and cellular kinetics that are not feasible experimentally. Here we study filament dynamics using two bi-ventricular 3-D high-resolution rabbit heart geometries, one with detailed fine structure and another without fine structure. We studied filament dynamics using anisotropic and isotropic conductivities, and with four cellular action potential models with different recovery kinetics. Spiral wave dynamics observed in isotropic two-dimensional sheets were not predictive of the behavior in the whole heart. In 2-D the four cell models exhibited stable reentry, meandering spiral waves, and spiral-wave breakup. In the whole heart with fine structure, all simulation results exhibited complex dynamics reminiscent of fibrillation observed experimentally. In the whole heart without fine structure, anisotropy acted to destabilize filament dynamics although the number of filaments was reduced compared to the heart with structure. In addition, in isotropic hearts without structure the two cell models that exhibited meandering spiral waves in 2-D, stabilized into figure-of-eight surface patterns. We also studied the sensitivity of filament dynamics to computer system configuration and initial conditions. After large simulation times, different macroscopic results sometimes occurred across different system configurations, likely due to a lack of bitwise reproducibility. The study conclusions were insensitive to initial condition perturbations, however, the exact number of filaments over time and their trends were altered by these changes. In summary, we present the following new results. First, we provide a new cell model that resembles the surface patterns of VF in the rabbit heart both qualitatively and quantitatively. Second, filament dynamics in the whole heart cannot be predicted from spiral wave dynamics in 2-D and we identified anisotropy as one destabilizing factor. Third, the exact dynamics of filaments are sensitive to a variety of factors, so we suggest caution in their interpretation and their quantitative analyses.

## Introduction

Sudden cardiac death resulting from ventricular fibrillation (VF) is the leading cause of death in industrialized countries. VF results from unstable reentrant waves of electrical activity throughout the heart (Winfree, 1994; Gray et al., 1995, 1998) that quickly leads to death if not terminated by an electric shock. Although high-resolution “optical mapping” has demonstrated organized electrical activity on the surface of small and large mammalian hearts in the form of spiral waves (Gray et al., 1995, 1998; Gray and Chattipakorn, 2005; Park and Gray, 2015), the detailed three-dimensional structure of such waves remains incompletely understood (Baxter et al., 2001; Fenton et al., 2018). Computer simulations of reentrant electrical waves in cardiac tissue continue to be an important tool to study the complex dynamics during fibrillation. Such simulations provide unique advantages, most notably the ability to study the relative roles of various factors such as heart geometry and cell kinetics as well as analyzing the fine resolution action potential dynamics throughout the entire heart, both of which are impossible to achieve experimentally.

Computer simulations have provided much insight into the detailed mechanisms of cardiac electrophysiological phenomena at the cell, tissue, organ, and whole-body levels (Noble, 1962; ten Tusscher et al., 2004; Trayanova and Rice, 2011; Viceconti and Hunter, 2016). The study of reentrant spiral waves in homogeneous sheets and cuboids using numerical and theoretical approaches have elucidated a variety of purported mechanisms for the phenomenon of “spiral wave breakup” which is thought to underlie VF (Gray, 2014). Reentrant spiral waves on the surface of the heart (or in a 2-D sheet) are identified as the instantaneous position of their tip where each tip is considered a singularity point or phase singularity (PS) (Gray et al., 1998). Spiral wave dynamics in 2-D homogeneous tissues can be quite varied as a result of the differences in cell model kinetics. Qu et al. (1999) studied the effects of restitution properties of major ionic currents on spiral wave behavior in 2-D tissue. In 3-D tissue reentrant “scroll” waves rotate around a line called a filament (Clayton and Holden, 2002b; Xie et al., 2004). Filaments and the resulting scroll waves can be of different shapes. For example, a stationary rotor in the 3-D space that spans from the epicardium to the endocardium, where the spiral wave is evident on both surfaces is the simplest form of a filament which is linear (Yamazaki et al., 2012). Filaments can also form different nonlinear shapes such as U, L, and O (Berenfeld and Pertsov, 1999). U-shaped filaments are manifest as a figure-of eight pattern on one surface and a breakthrough pattern on the opposite surface, while O-shaped filaments, which are the form of a ring, reside completely within the myocardium so that no surface PSs will be visible. Theoretical studies on 3-D tissue have identified unique properties of filaments including filament tension (Panfilov and Rudenko, 1987) that determines whether filaments grow or shrink (Biktashev et al., 1994). For healthy, highly excitable cardiac tissue it is thought that filaments have positive tension and tend to shrink and for ischemic tissue where the excitability has decreased, it is believed that filaments have negative filament tension where the filaments grow, and that growing filaments play a primary role in VF. This theory has been challenged by further theoretical investigations where highly excitable tissue even showed negative filament tension (Alonso and Panfilov, 2008). Fenton and Karma (1998) introduced another uniquely 3-D form of scroll wave instability which results from high transmural wave front curvature due to the twisting of fibers across the ventricle wall. These *twistons* can propagate along the filament and *break off* to form new filaments.

Filament dynamics in 3-D slabs (Clayton and Holden, 2002a) and whole heart models (Clayton and Holden, 2004; Arevalo et al., 2007; Ten Tusscher et al., 2007, 2009; Clayton, 2008; Bishop and Plank, 2012) are quite complex and varied. A detailed modeling study on a canine whole heart filament analysis (Clayton and Holden, 2004) studied the number of filaments, their life time, initiation, and termination in both the left ventricle (LV) and right ventricle (RV) walls following the initiation of reentry. They studied how these metrics are affected by membrane kinetics and geometry. Clayton and Holden (2004) used a three variable Fenton-Karma model (Fenton and Karma, 1998) which exhibited spiral wave breakup in 2-D (Clayton et al., 2006) to represent the ion channel dynamics in the canine whole heart (Clayton and Holden, 2004). There are also studies using a modified Fitzhugh-Nagumo model (Panfilov and Hogeweg, 1993) to describe the spiral wave behavior using a canine whole heart and 2-D tissue (Gray et al., 1998; Panfilov, 1998). Both studies show a single spiral wave initiated on a 2-D tissue breaking into multiple spiral waves. Spiral breakup starts close to the center of the initial spiral and spreads out over the entire tissue. In the canine whole heart, the spiral wave breakup is similar to 2-D wave break (Clayton and Holden, 2004). The original Luo-Rudy model (Luo and Rudy, 1991) has been modified and used in pig whole heart and 2-D tissue to study scroll wave dynamics (Majumder et al., 2016) and reentrant spiral waves (2-D) (Qu et al., 1999). Majumder et al. (2016) studied the effect of conduction inhomogeneities and ionic inhomogeneities on scroll wave dynamics on the pig whole heart. They found that small scale conduction inhomogeneities and large scale ionic inhomogeneities affect scroll wave dynamics substantially. Arevalo et al. (2007) studied the role of intrinsic heterogeneities in action potential. They found that action potential duration (APD) heterogeneities leads to increased complexity of VF organization with a two-fold increase in the number of filaments. These numerical studies provide unique information, are useful in the design of experimental studies, and provide a comprehensive “scaffold” for which to help translate the results of benchtop studies and understand reentrant arrhythmias in the clinical setting.

Although computer simulations allow the ability to deconstruct the structural and functional elements of fibrillation, translating the results from homogeneous tissue with simple geometries to the whole heart has proven to be problematic. Xie et al. (2004) performed simulations in a dog ventricle with fibers and concluded that cellular kinetics are a major factor controlling instabilities. Similarly, Clayton and Holden (2004) found that scroll wave breakup in the same canine geometry was related to steep APD restitution and that the total number of filaments increased faster for initiation in the thicker left ventricle (LV) compared to the thinner right ventricle. Pravdin et al. (2013) have developed an elegant analytical representation of the LV geometry and fibers which enables a systematic study of 3-D scroll wave dynamics in somewhat realistic ellipsoid geometries. The final position of the scroll waves were mainly determined by wall thickness, although anisotropy attracted the filament to the LV apex (Pravdin et al., 2013). In addition, an apical-base heterogeneity resulted in an increased scroll wave drift velocity and a shift toward the region of maximum action potential duration (Konovalov et al., 2016).

The present paper follows on from two recent papers (Bishop and Plank, 2012) Pathmanathan and Gray (2015) analyzing filament dynamics on an anatomically detailed rabbit ventricular model based on magnetic resonance imaging data (Bishop et al., 2010). This geometrical model is unique in that it contains high level of detail including large intramural vessels, papillary muscles, and trabeculae. A corresponding mesh of the same geometry was also developed by “smoothing” these features (Bishop et al., 2010). In this study Bishop and Plank (2012) concluded that fine structure does not play a role in VF dynamics by correlating filament locations with geometric structures. Our previous study by Pathmanathan and Gray (2015) used three parameterizations of a recently developed cell model (Gray and Pathmanathan, 2016) to simulate reentry in the anatomically detailed geometry (only). In 2-D one of the cell models exhibited stable reentry, another cell model exhibited spiral-wave breakup and the other exhibited spiral wave meandering behavior. Surprisingly, all three cell models exhibited spiral-wave breakup and VF-like activity in the whole heart (with fine structure). Pathmanathan and Gray (2015) suggested that anatomical structure may play an important role in the initiation and the maintenance of VF contrary to the conclusions of Bishop and Plank (2012). We believe that this apparent discrepancy in conclusions is methodological, because Pathmanathan and Gray (2015) came to their conclusion by studying filament dynamics while (Bishop and Plank, 2012) correlated filament locations with geometrical structures. Here we provide new simulations designed to extend these two previous studies (Bishop and Plank, 2012; Pathmanathan and Gray, 2015) by performing simulations using the cell models in Pathmanathan and Gray (2015) using the “smoothed” (without blood vessels, papillary muscles, and trabeculations) bi-ventricular rabbit geometry of (Bishop et al., 2010). These new simulation results provide a full complement that allows a direct comparison of the effects of fibers (anisotropy), structure, and cell dynamics on simulated VF in the rabbit heart.

## Methods

This paper extends our previous work (Pathmanathan and Gray, 2015), and most of the below methods reproduce methods used in this previous paper. Simulations were carried out by solving the monodomain equations governing electrical activation and propagation in excitable tissue (Keener and Sneyd, 1998). The monodomain equation is

(1) $$\begin{array}{r} \chi \left(C_{m}\;\frac{\partial V}{\partial t}+I_{ion}\left(\text{u},V\right)\right)-\;\nabla .\;\left(\sigma \nabla V\right)=0, \end{array}$$

where *V* is the transmembrane voltage, ***u***is a vector of cellular state variables (***u*** = *[m, h], m* and *h* defined below), χ = 1,400 cm^−1^ is the surface-area-to-volume ratio, and C_m_ = 1.0 μF cm^−2^ is the capacitance per unit area. Both anisotropic and isotropic simulations were performed. For anisotropic simulations the conductivity tensor σ was set as χC_m_ D_L_ in the fiber direction and χC_m_ D_T_ in the cross-fiber directions, where D_L_ = 0.001 cm^2^ ms^−1^ and D_T_ = D_L_/9, chosen to match fiber and cross-fiber conduction velocities on the epicardial surface of the rabbit heart (Schalij et al., 1992). For isotropic simulations σ was chosen to be a scalar with value 0.466 mS cm^−1^ as done previously (Pathmanathan and Gray, 2015) which preserves the surface area in any plane parallel to the fiber direction; by doing so the heart surface area is conserved but transmural thickness is decreased. The monodomain equations were solved using the finite element method using the CHASTE software package (Mirams et al., 2013). The CHASTE software package has been proven to be a powerful tool in computational cardiac electrophysiology over time with rigorous verification tests done on it (Niederer et al., 2011; Pathmanathan et al., 2012; Pathmanathan and Gray, 2014).

We used the parsimonious rabbit cell model developed by Gray and Pathmanathan (2016). This cell model consists of two ionic currents (*I*_*Na*_ and *I*_*K*_), three variables and eleven parameters. The sodium current *I*_*Na*_ activates rapidly upon depolarization and is based on the Hodgkin-Huxley sodium current model with activation variable *m* and inactivation variable *h*. The second current that describes time-independent inward-rectifying repolarization ($I_{K}=\;g_{K}\left(V-\;V_{rest}\right)e^{-\beta \left(V-\;V_{rest}\right)},\;V_{rest}=\;-83\;mV$) is a phenomenological repolarization current based on the experimental data for a whole rabbit heart (Gray et al., 2013). The total ionic current (*I*_*ion*_) is the sum of *I*_*Na*_ and *I*_*K*_ given as:

(2) $$\begin{array}{r} I_{ion}=\;I_{Na}+\;I_{K} \end{array}$$

For *I*_*Na*_ parameter values see (Gray and Pathmanathan, 2016) Table 1 and erratum. Four cell models with different *I*_*K*_ parameters were constructed. Cell models P1-P3 were identical to the three cell models studied in (Pathmanathan and Gray, 2015); they use *g*_*K*_ = 0.5 μA cm^−2^ mV^−1^ and different values for β (P1: β = 0.03 mV^−1^; P2: β = 0.035 mV^−1^; P3: β = 0.04 mV^−1^). A fourth cell model (P4: β = 0.035 mV^−1^, *g*_*K*_ = 0.3 μA cm^−2^ mV^−1^) was also introduced to better replicate VF in the isolated rabbit heart. Parameter sets P1-P3 were originally chosen because they exhibited varied spiral wave dynamics in two-dimensional (2-D) isotropic sheets (**Figure 2A**) (P1–stable reentry, P2–spiral wave breakup, P3–meandering behavior) (Pathmanathan and Gray, 2015). In 2-D tissue, model P4 also shows meandering behavior (**Figure 2A**).

Simulations were run on the same two high resolution computational meshes of the rabbit ventricles developed by Bishop et al. (2010). The first is a complex fine-scale anatomical mesh featuring blood vessels, papillary muscles, and trabeculations (Bishop et al., 2010). This highly-detailed anatomical mesh was generated directly from high-resolution (25 μm isotropic) magnetic resonance (MR) data. The second simplified mesh (the “smoothed mesh”) was also generated from the same MR data set, but all endocardial structures were removed, and intramural cavities filled during the mesh generation process (Bishop et al., 2010). Both the complex and smoothed meshes consist of nearly 4 million nodes and 24 million tetrahedral elements and have an average edge length of ~125 μm.

We carried out the simulations using the same methods as (Pathmanathan and Gray, 2015) including the same stimulation protocol used to obtain VF, in which we apply an initial stimulus (S1) near the apex followed by a second stimulus (S2) applied to a large ellipsoidal region on the lateral posterior wall of the left ventricle timed to overlay with the apex to base repolarization wave. Once reentrant activity had been induced, filaments were computed using the same methods as described in Pathmanathan and Gray (2015). In addition to computing filaments, phase singularity density was calculated using a 1.5 × 1.5 cm square area on the epicardial surface of the posterior regions described in Iyer and Gray (2001).

Simulations were run on a desktop computer with 128 GB RAM and with two Intel Xeon E5-2650 V3 processors with each having 10 cores for a total of 20 cores. With the hyperthreading capability of the processors giving us 40 threads, we used only 20 threads for each simulation that takes around 4 h of computational time for a simulation time of 2 s.

## Results

**Figure 1:**
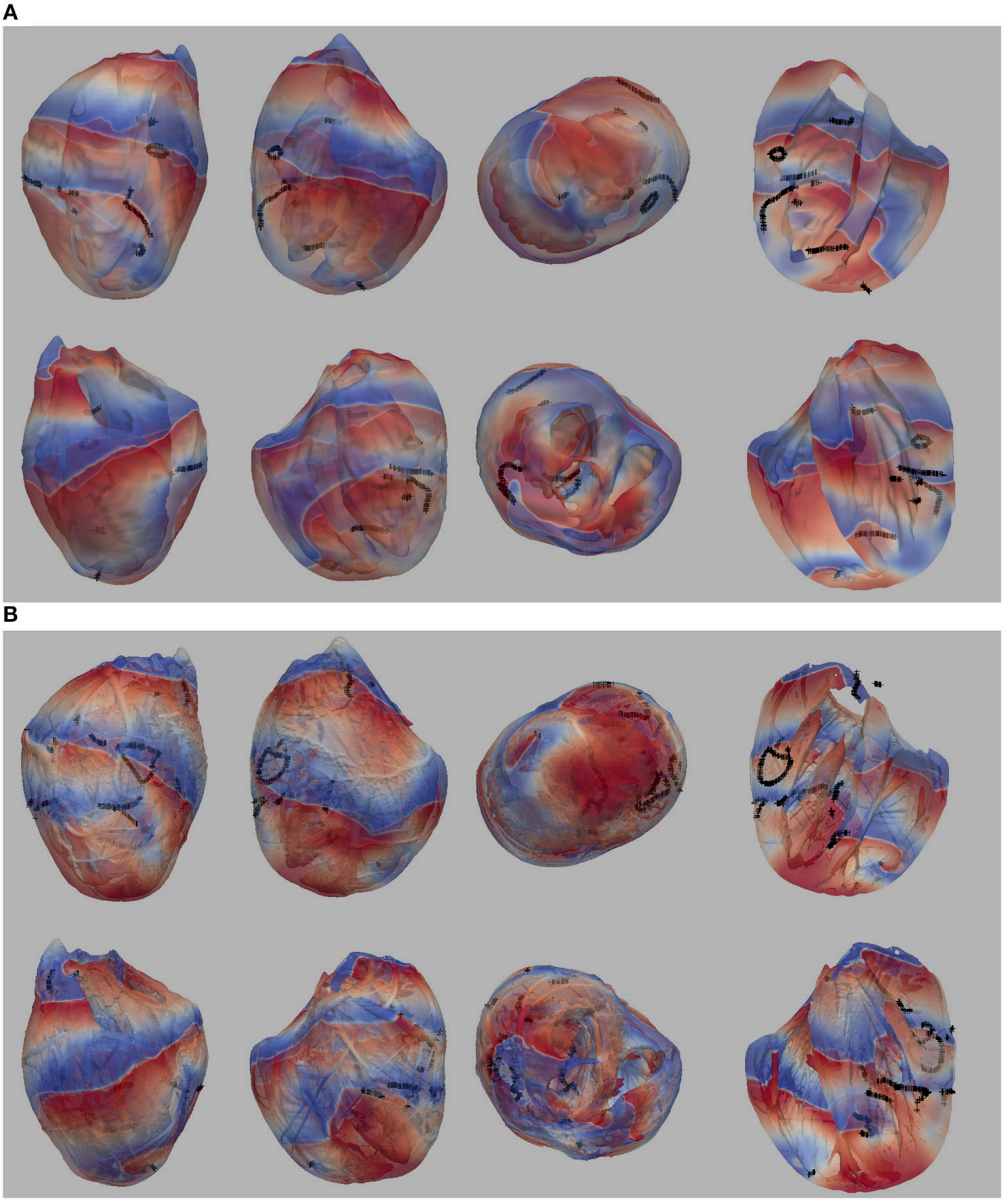
**(A)** Multiple views of simulated fibrillation for simulation P2_A_nS (cell model P2, anisotropic conductivities, mesh with no fine-scale structure) at 320 ms after initiation. **(B)** Multiple views of simulated fibrillation P2_A_wS (cell model P2, anisotropic conductivities, mesh with fine-scale structure) at 320 ms after initiation. Transmembrane potential is represented with a blue-red color map such that blue corresponds to −83 mV and red to +20 mV. Filaments are shown as thin black curves.

In our previous work (Pathmanathan and Gray, 2015) we analyzed reentrant activity that arose in models P1-P3 using the mesh containing fine-scale structure and both isotropic and anisotropic conductivity tensors (six combinations). Here, we performed the same simulations for models P1-P4 using the smoothed mesh; in addition, we carried out (P4) and repeated (Benureau and Rougier, 2018) (P1-P3) simulations in the fine structured mesh (Pathmanathan and Gray, 2015). Overall, we analyzed the dynamics of spiral waves in isotropic 2-D sheets and scroll waves in bi-ventricular heart geometries with fibers [anisotropic (A)], and without fibers [isotropic (I)], as well as on the meshes with structure (wS) and without structure (nS). We refer to the 16 possible biventricular simulations using these acronyms [e.g., the isotropic simulations using cell model P2 on the smoothed (no structure) geometry is “P2_I_nS”]. A snapshot of activity (320 ms after initiation) from multiple views is shown for simulation P2_A_nS and P2_A_wS in Figure 1 (the corresponding figures for the other models are shown in the Supplementary Material).

From Figure 1 we can see the complex VF pattern and filament behavior due to the sustained activity. Linear filaments were visible whenever a scroll wave spans from epicardium to the endocardium with spiral waves visible on both surfaces. We observed O-shaped filaments in the form of a ring shape, that reside completely within the myocardium so that no surface PS were visible. U-shaped filaments were also found which were manifest as a figure of eight pattern on one surface and a breakthrough pattern on the opposite surface. In summary, simulated VF activity visible from the movies (SMovie 10) demonstrate complex filament behavior associated with VF.

### 2-D Spiral Wave Dynamics

**Figure 2:**
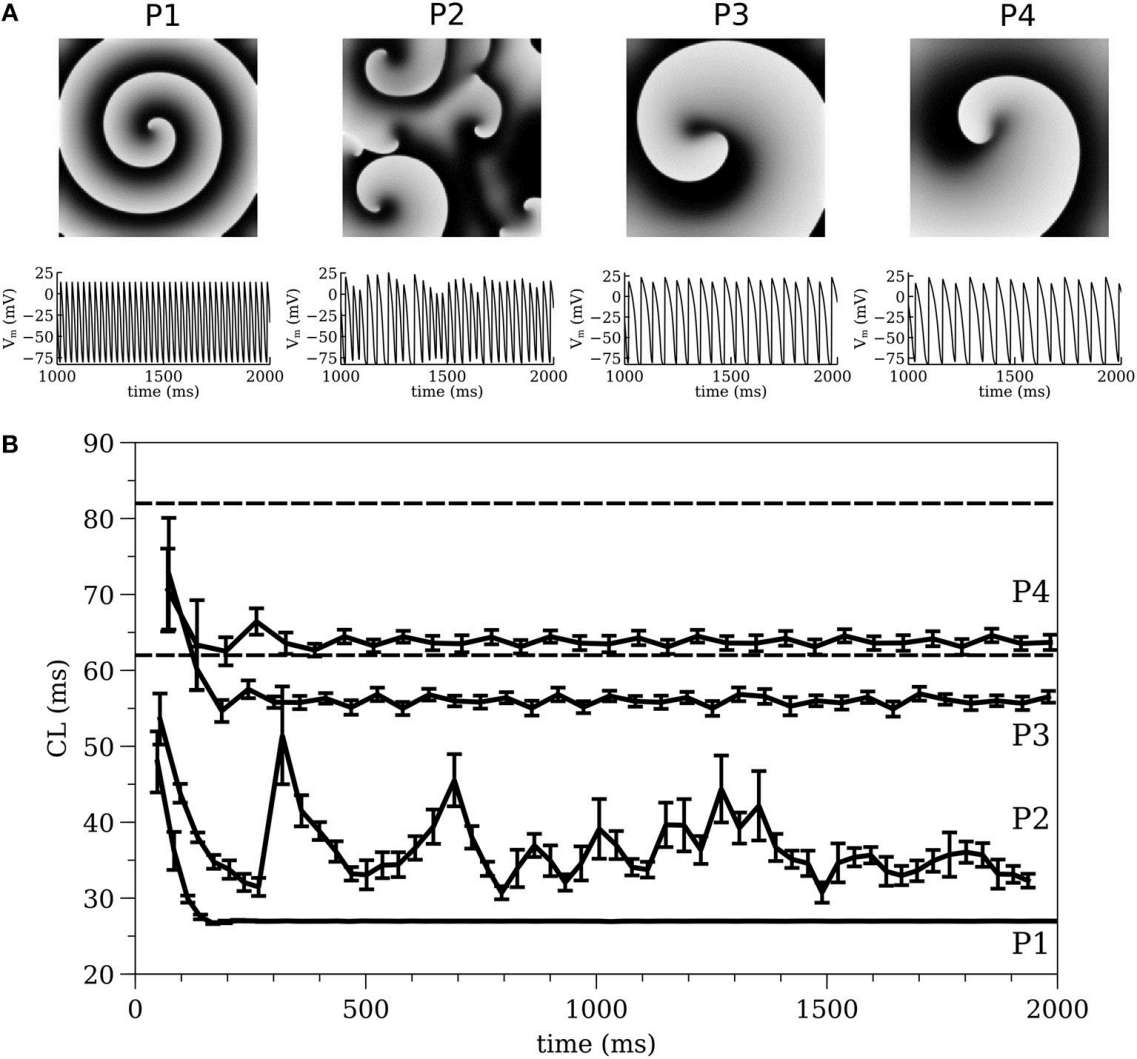
**(A)** 2-D spiral wave dynamics with corresponding action potential traces and **(B)** Average cycle length computed from 2-D simulations, for all models of P1, P2, P3, and P4. The dashed lines indicate the range of CL measured in the isolated rabbit heart during VF experimentally by Chorro et al. (2000).

The spiral wave dynamics in 2-D simulations are shown for models P1-P4 in Figure 2A. Stable reentry around a small (~0.01 cm^2^) circular core was observed for P1, while spiral wave breakup occurred for P2 and meandering spiral wave resulted for P3 and P4 [the meandering region was smaller (P3–1.1 × 1.1 cm and P4–0.9 × 0.9 cm) and the cycle length (CL) longer for P4 (~65 ms) compared to P3 (~55 ms)]. The dynamics of average CL (computed from 16 sites) for all spiral wave simulations are shown in Figure 2B for two seconds immediately following initiation. The dashed lines indicate the range of CL measured in the isolated rabbit heart during VF by Chorro et al. (2000). The CL dynamics for the stable (P1) and meandering (P3 and P4) spiral waves stabilized after a decrease within the first 200 ms while the CL dynamics or P2 was quite different with large fluctuations. The mean values for the four parameter sets were different, with P1 having the smallest value (and the smallest variance).

### Cycle Length (CL) Dynamics

**Figure 3:**
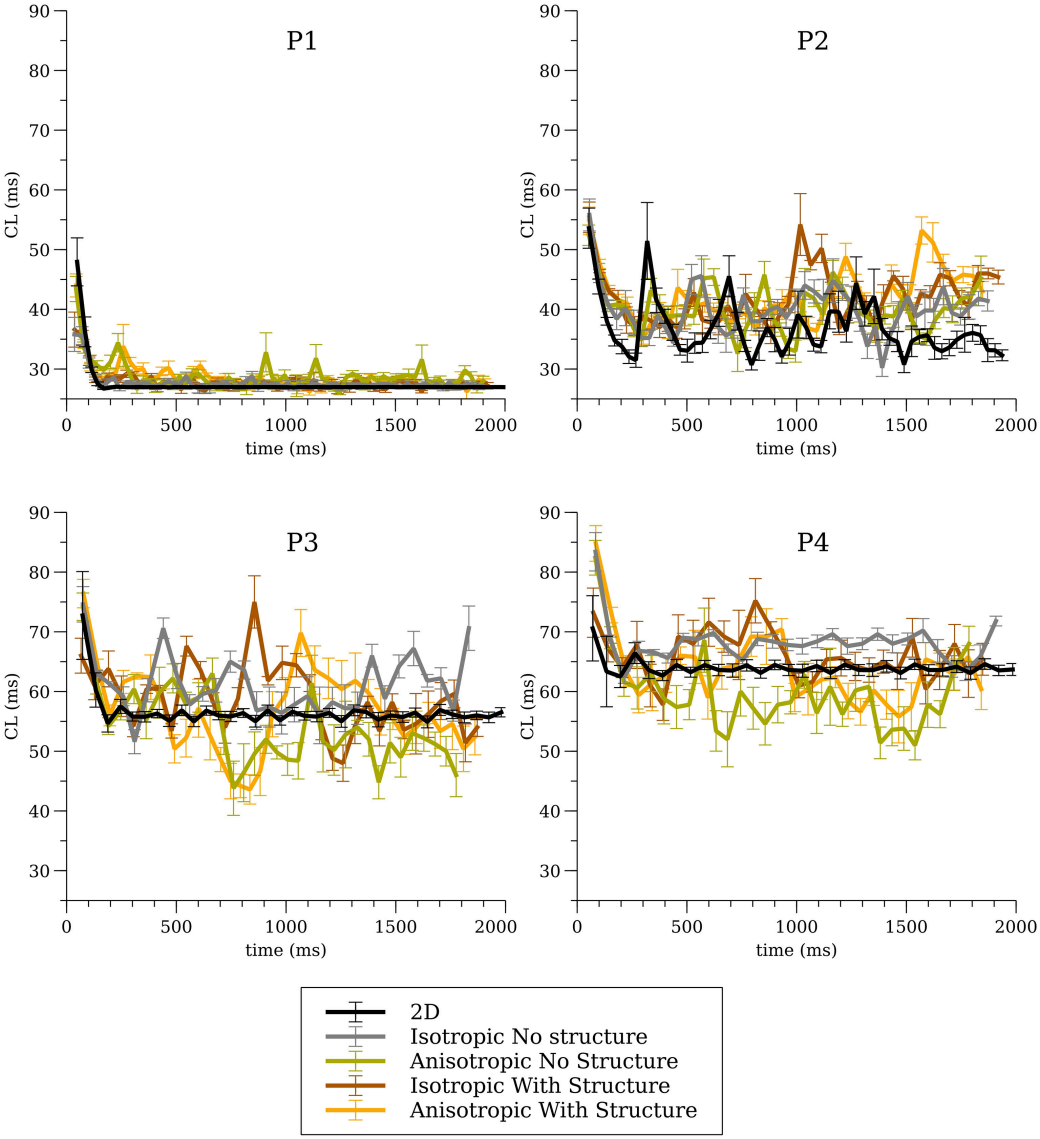
Average cycle length (CL) for the whole heart simulations for all four cell models for all conditions.

**Figure 4:**
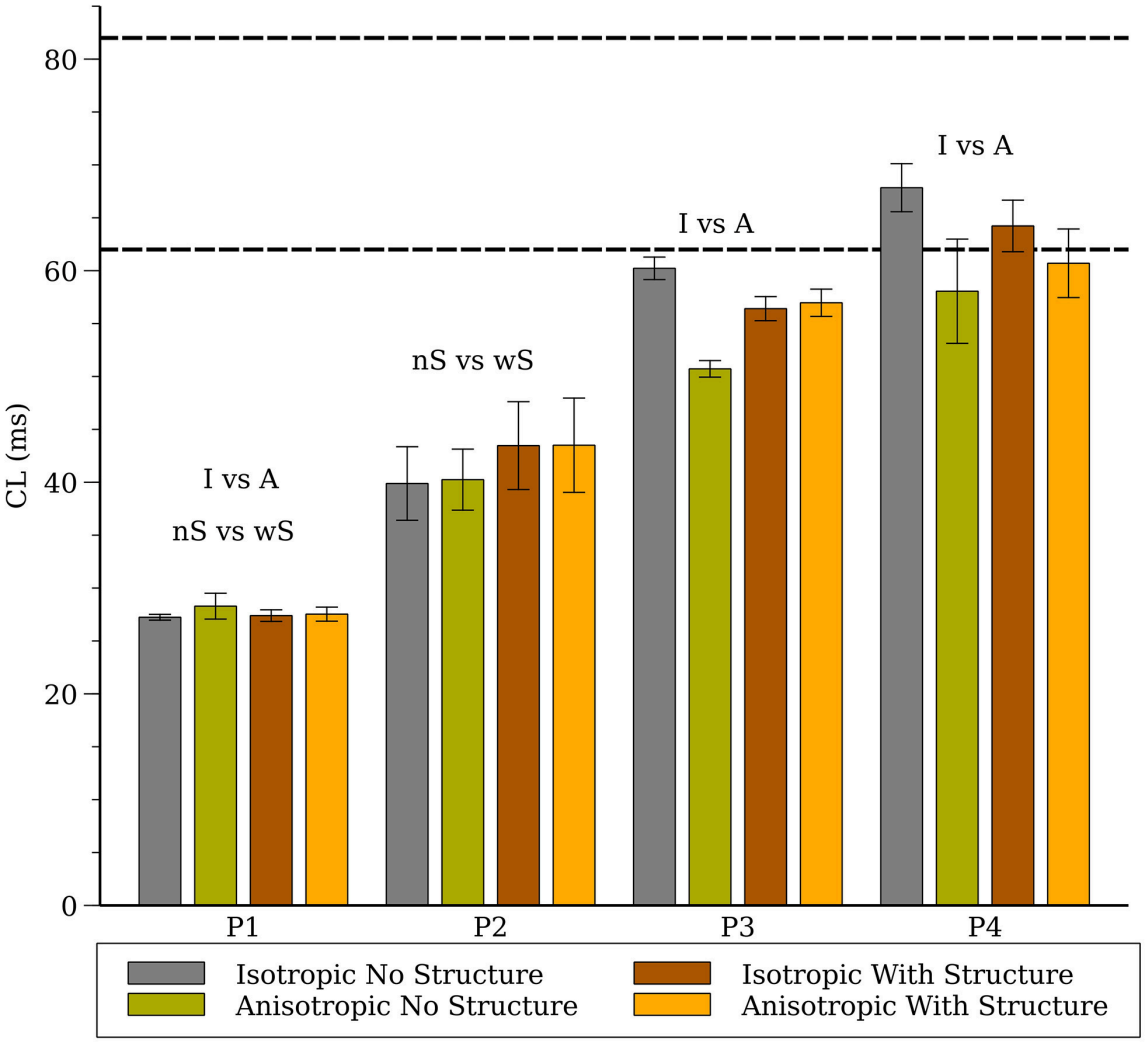
Average cycle length (CL) for the whole heart spiral wave simulations for all four cell models. The dashed lines indicate the range of CL measured in the isolated rabbit heart during VF experimentally by Chorro et al. (2000). The labels (“I vs. A”, “nS vs. wS”) state whether inclusion of anisotropic conductivity, and/or inclusion of structure in the mesh, statistically affects cycle length.

The average CL (computed from 16 sites) for the 3-D whole heart simulations are shown in Figure 3 for the first two seconds of activity; the values for 2-D spiral waves from Figure 2B are replotted here for comparison as thick black lines. To analyze the influence of structure and fibers we performed two-way analysis of variance (ANOVA) on these CL values between one and two seconds for the four models as shown in Figure 4. This analysis revealed that the CL between one and two seconds was influenced by structure for models P1 and P2 and was influenced by fibers for P1, P3, and P4.

### Filament Dynamics

**Figure 5:**
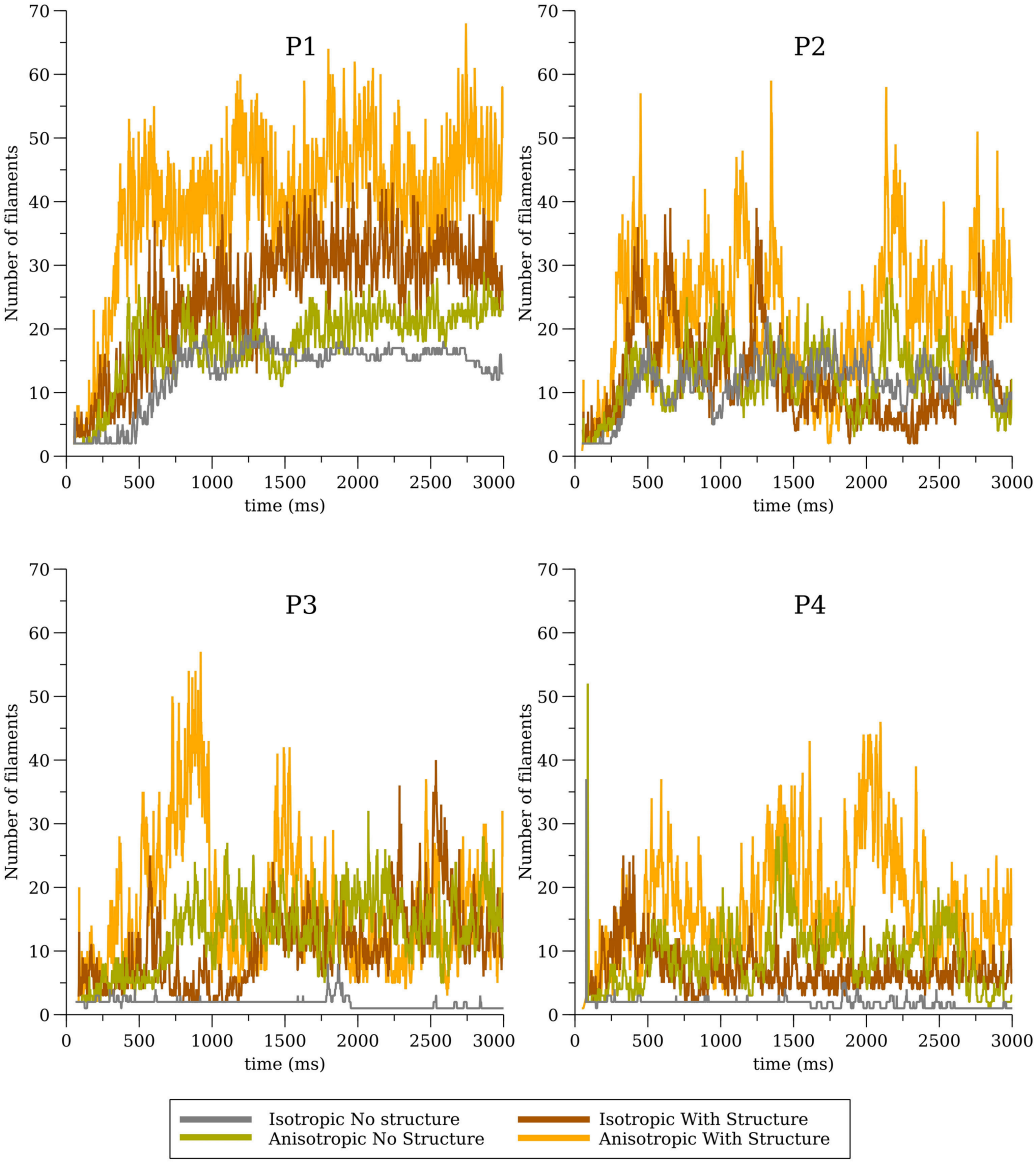
Filament dynamics in the whole heart for all four cell models (P1, P2, P3, and P4) with and without structure and with and without anisotropic conductivities.

**Figure 6:**
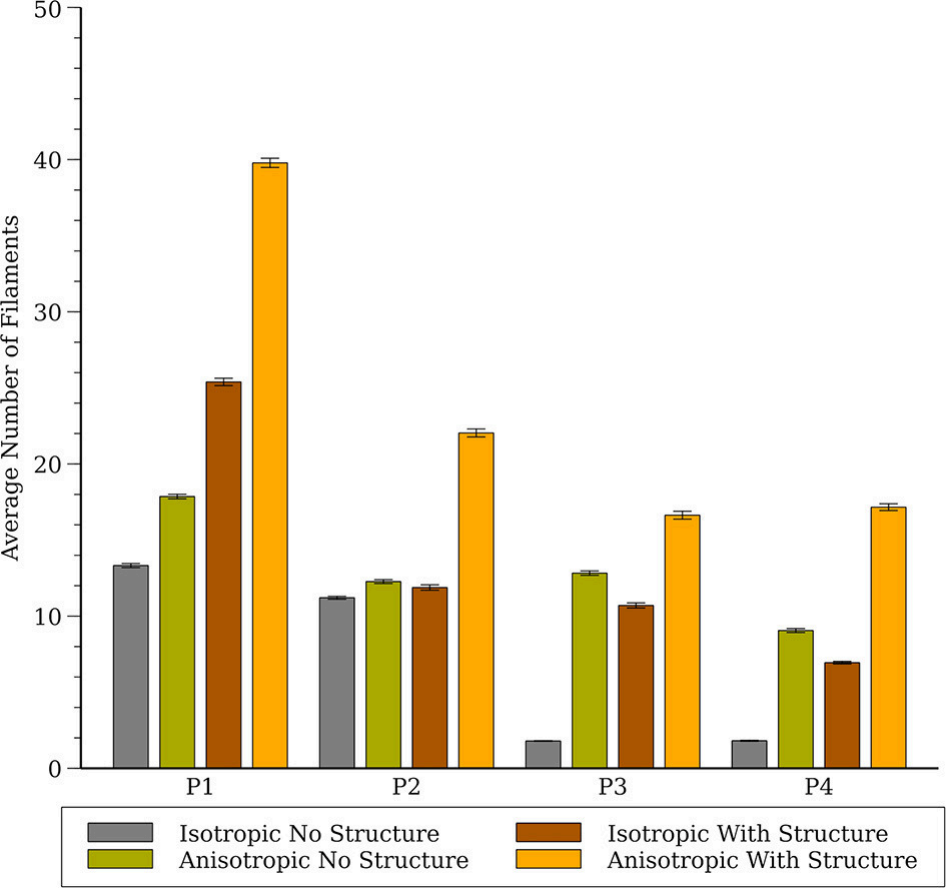
Average number of filaments in the whole heart for all four cell models (P1, P2, P3, and P4) with and without structure and with and without anisotropic conductivities.

**Figure 7:**
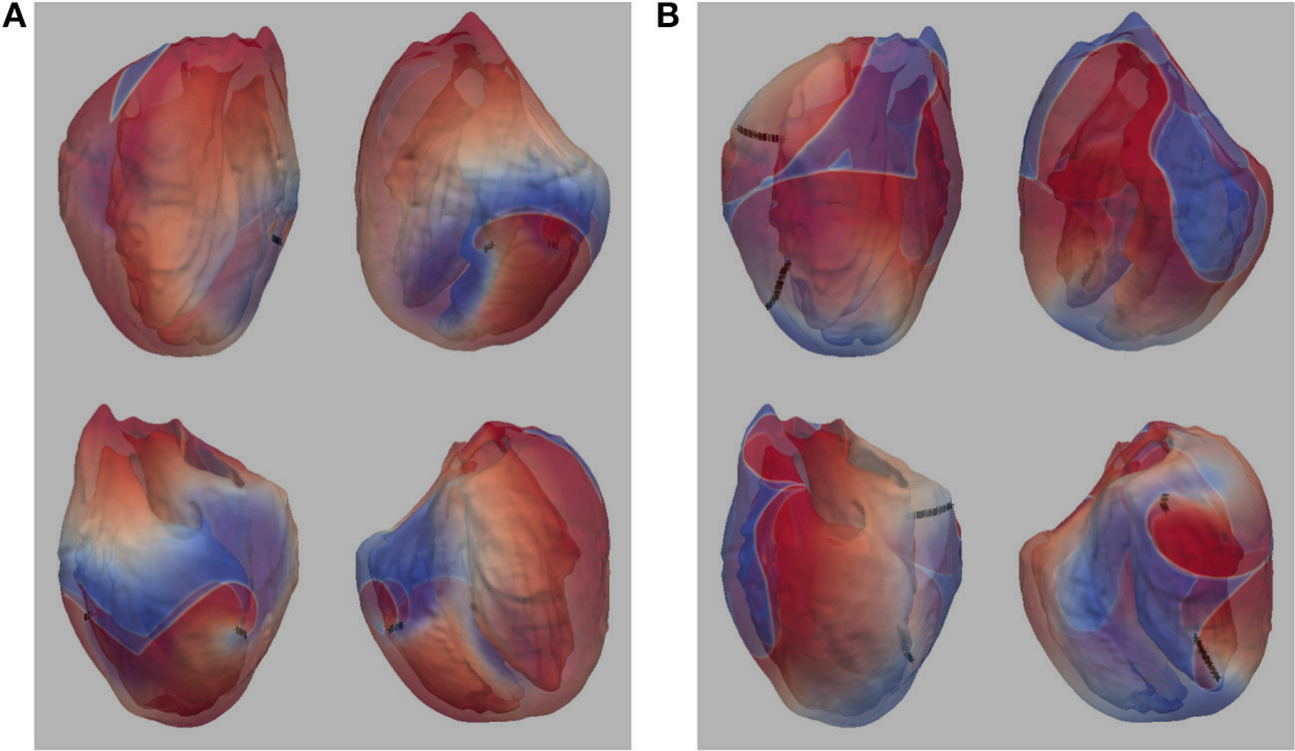
**(A)** Snapshot of stable activity in P3_I_nS **(B)** Snapshot of stable activity in P4_I_nS. Filaments are shown as thin black curves.

**Figure 8:**
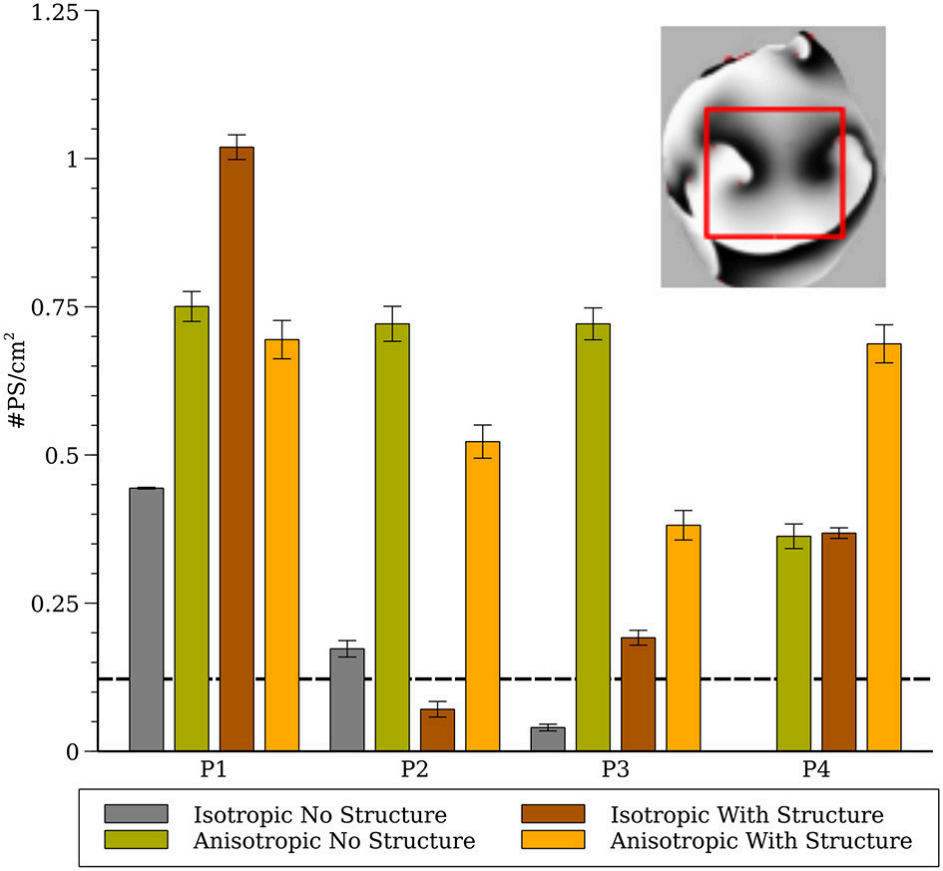
Phase singularity density in the whole heart for all four cell models (computed from a 1.5 × 1.5 cm region). The inset shows the region of the posterior surface where the phase density was calculated. The dashed line indicates experimental measurements of PS density from rabbit hearts (Gray et al., 1998; Harada et al., 2011).

The number of filaments for the first 3 s of activity for all four models are shown in Figure 5. The number of filaments exhibit similar trends for isotropic and anisotropic conditions for P2 and P1 models; the number of filaments increased during the first second and then remained near 15–20 with some fluctuations. For the isotropic no structure (but not anisotropic) P3 and P4 simulations, however, the average number of filaments remained very low (~2) (see Figure 6). Figure 7 illustrates a snapshot of stable activity with a low number of filaments (see Figure 5) for the P3 and P4 isotropic no structure simulations. For P3_I_nS (panel A) there are no filaments manifest on the posterior surface (hence zero PS density in Figure 8) but the stable arrhythmia was maintained via a stable scroll wave on the posterior side wall. The simulations for P4_I_nS (panel B) evolved into a stable arrhythmia with a low number of filaments. This filament behavior is described earlier where two linear filaments spanning from epicardium to endocardium is visible on both surfaces on either side of the ventricle. Supplementary files for movies (SMovies 3, 4) show these stable patterns in greater detail.

To compare our simulation results to experimental results from rabbits we generated phase maps from the epicardial posterior surface and computed the number of phase singularities in a 1.5 × 1.5 cm region and computed the average phase singularity density between 1 and 2 s as shown in Figure 8. The dashed line indicates experimental measurements from rabbit from (Gray et al., 1998; Harada et al., 2011).

### Sensitivity of Simulation Results to System Configuration and Initial Conditions

**Figure 9:**
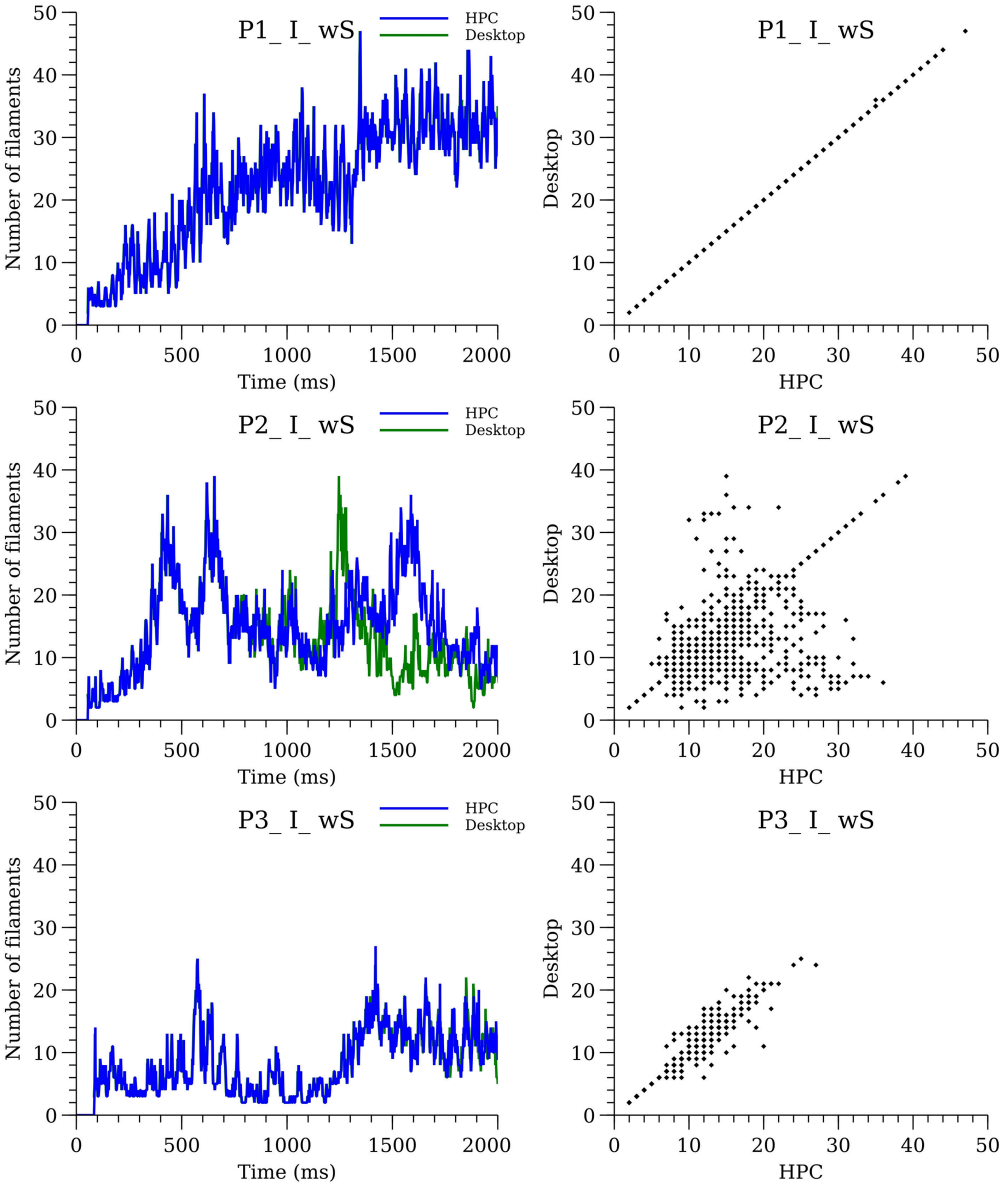
Sensitivity of filament dynamics to system configuration for isotropic simulations with structure. The plots on the left-hand side show the number of filaments over time for desktop (green line) and HPC (blue line) configurations. The plots on the right show the divergence between the two configurations.

Six of the whole-heart simulations (wS, P1-P3, I, and A) performed in Pathmanathan and Gray (2015) were repeated. Previous simulations were run on a high-performance computing (HPC) system using CHASTE; present simulations were run on a desktop with a more recent version of CHASTE (CHASTE version 2017_1). We observed that in some cases the spatial surface patterns, and therefore the number of filaments, were different between the present and previous results if the simulations are run for long enough—see Figure 9 (isotropic; anisotropic in Supplementary Material). The precise divergence of filament dynamics varied with cell model and condition, with some simulations being nearly identical for 2 s (SMovies 5, 7, and 13).

**Figure 10:**
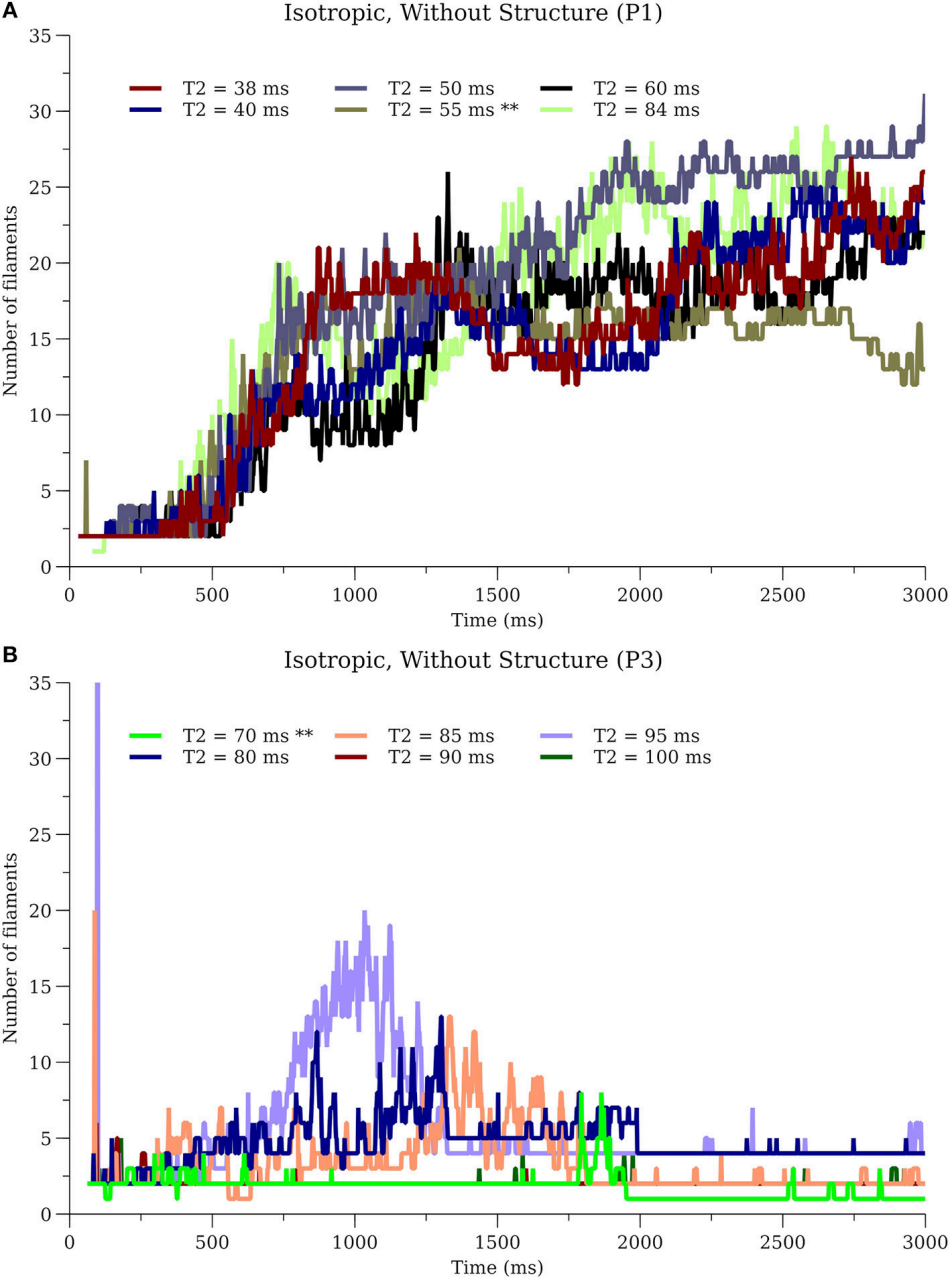
Sensitivity of filament dynamics to initial conditions. **(A)** P1_I_nS **(B)** P3_I_nS. ^**^ in T2 values indicate the simulations used for results in other sections.

To further investigate sensitivity, simulations for P1_I_nS and P3_I_nS were performed on our desktop computer while varying the time of the S2 stimulation covering the range of the vulnerable period. Simulations for P1_I_nS were relatively insensitive to the timing of the S2 while simulations for P3_I_nS exhibited sensitivity to the S2 timing, see Figure 10.

## Discussion

Although it is reasonable to extrapolate simulation results from the numerous studies of spiral wave dynamics in 2-D to the whole heart, and in some situations this approach seems justified (Xie et al., 2004), as we have showed previously, fibrillatory activity in the whole heart can occur even for cell models that exhibited stable or meandering spiral wave dynamics in 2-D (Pathmanathan and Gray, 2015). Here we provide the following new results. First, by completing the full complement of 16 combinations of cell model, fibers, and level of structural detail we conclude that fine scale structure and anisotropy acts to destabilize reentrant waves. The only simulations which exhibited stable reentry were for isotropic hearts without fine structure. In addition, even for isotropic hearts without structure one cell model (P1) that exhibited stable spiral waves in 2-D resulted in VF-like activity for all conditions as shown in Figure 5 which we attribute to the short wavelength in this model (Park and Gray, 2015). Second, we present a novel cell model (P4) which results in VF-like activity that is quantitatively similar to experimental results. Given that this P4 model is very simplistic, it is not appropriate for the study of many cardiac phenomena such as APD restitution, nevertheless, it is a “minimal” model that can be used to simulate VF in the rabbit.

We demonstrated previously that negative filament tension was not responsible for the scroll wave instabilities, but we could not rule out numerical artifacts resulting from the fine scale heterogeneities (Pathmanathan and Gray, 2015). The results presented here suggest that such artifacts are unlikely because models P1 and P3 also exhibited fibrillation-like activity in the smoothed mesh with the exception of P3_I_nS which demonstrated stable activity (ventricular tachycardia) between 2 and 3 seconds as shown in Figure 7. Overall, our results strongly demonstrate that macroscopic heterogeneities in ventricular structure including fibers are sufficient to destabilize 3-D reentrant waves. Simulation P4_I_nS stabilized into non-fibrillatory (ventricular tachycardia); however, models P3 and P4, despite exhibiting meandering behavior in 2-D, exhibited VF-like activity in the presence of structure and fibers. As shown in Figure 7, the stable filaments in P4_I_nS were not manifest on the portion of the epicardial posterior surface used to compute PS density as seen Figure 8. Interestingly, P2_I_wS showed a smaller PS density compared to other P2 simulations (see Figure 8) although the filament dynamics were similar (see Figure 5). The results in Figure 8 when interpreted in light of the sensitivity of the simulations suggest that PS density quantification may not be appropriate unless the whole surface is used for the computations as can be achieved with panoramic imaging (Lou et al., 2008; Gloschat et al., 2018). Optical mapping experiments on swine hearts by Chen et al. (2000) and Kay et al. (2006) give us important insights in to the life span of PSs. Though we have not performed an analysis of life span of PSs in our simulations, mainly due to the technical difficulty in obtaining compound filaments from a 3-D structure, we would expect to have a high number of short-lived fragments. In addition, as expected fine scale structure increased the number of filaments and we presume also the number of short-lived wave fragments.

The results from parameter sets P3 and P4 were most similar to experimental results from VF in the isolated rabbit heart in regard to CL (Figure 4) and PS density (Figure 8). Both P3 and P4 exhibited meandering spiral wave dynamics in 2-D, but fibrillation-like activity in the whole heart. For both these parameter sets the fibers influenced CL as shown in Figure 4. While the CL and PS densities values from P3 and P4 were similar to experimental results (Gray et al., 1998; Chorro et al., 2000; Harada et al., 2011), there were differences among conditions and given the sensitivity of simulation results it is unclear how exactly to compare them to experimental data in a rigorous statistical manner. It should be noted that while VF is observed in the whole rabbit heart experimentally (Chorro et al., 2000; Samie et al., 2000), only monomorphic and polymorphic reentry is observed when only a surviving epicardial rim remains after cryoablation (Schalij et al., 2000; Kodama et al., 2005) which is consistent with the simulation results of P3 and P4. It should be appreciated, however, that the majority of arrhythmia studies in the isolated rabbit heart have utilized voltage-sensitive dyes that provide unique insights into the corresponding spatial patterns. These optical mapping studies are carried out using agents that disrupt electrical-mechanical transduction which have been shown to alter the dynamics and duration of arrhythmias (Samie et al., 2000; Banville and Gray, 2002; Lou et al., 2011; Park et al., 2014). Nevertheless, filament analysis on human heart (Ten Tusscher et al., 2007, 2009) have shown that the number of filaments in the human is similar compared to the rabbit heart with structure in our P4 cell model that show VF-like dynamics quantitatively similar to experiments. In addition, due to theoretical considerations regarding wavelength, it has been suggested that VF in the rabbit is most similar to humans compared to pig and dog (Panfilov, 2006).

In addition to the 16 combinations of cell model (P1-P4), fibers (A or I), fine structure (wS or nS) we analyzed the sensitivity of simulation results to computer architecture and initial conditions (i.e., S2 timing). While in some cases, the precise filament dynamics were identical (for system configuration only) or qualitatively similar (see Figure 10A), in other cases the differences were notable (see middle of Figures 9, 10B). Since CHASTE is supported by a comprehensive suite of unit and regression tests [including tests that the monodomain equation is solved correctly and at the expected rate of convergence(Pathmanathan and Gray, 2014)], and because the divergence only occurs after hundreds of thousands of timesteps, it is reasonable to conclude that the eventual numerical differences in Figure 9 are due to differences in the system configuration (HPC vs. desktop) rather than software version or user error. Specifically, lack of bitwise reproducibility, which is not unexpected in parallel codes (Balaji and Kimpe, 2013), eventually leads to divergence of the activity. We believe that our results argue against detailed quantitative interpretation of filament dynamics in whole heart simulations, although statistical analyses of multiple simulations may be justifiable. In hindsight, this sensitivity is not surprising because of the well-known sensitivity when numerically integrating highly non-linear equations over long periods of time. While the differences due to system configuration could be eliminated by demanding that the cardiac simulation software exhibits bitwise reproducibility across different computer architectures (a very strong test that is not exhibited in any cardiac solver, as far as we are aware), the model would still be sensitive to changes in S2 stimulation time (see Figure 10), etc.

There are several limitations of our study that should be considered when interpreting our results. We did not incorporate any APD heterogeneity in our study, though a previous study (Arevalo et al., 2007; Dosdall et al., 2007) have shown that VF is affected by APD differences in the right and left ventricles. The study by Arevalo et al. (2007) shows that there are around 17 filaments in the whole rabbit heart with heterogeneous APD [Figure 8 of Arevalo et al. (2007)] for anisotropic smooth structure, which compares favorably to our simulations (see anisotropic results without structure in Figure 6). Based on Arevalo's study we expect that a similar RV-LV gradient of APD would increase the average number of filaments, however it is unclear how other APD gradients (e.g., transmural) would affect our results. The effect of the Purkinje system on VF is another complexity that we did not incorporate in our simulations. Experimental studies by Dosdall et al. (2007) in the pig and simulation studies by Berenfeld and Jalife (1998) and Behradfar et al. (2014) have shown some effects of Purkinje system on the dynamics of VF; however their exact role in VF initiation and maintenance remains largely unknown. We believe that the inclusion of both APD gradients and the Purkinje system would likely increase the complexity of our simulations results leading to a larger number of filaments. We chose to use a novel cell model because there is no validated model for rabbit VF. Previous cell models have been developed to represent other important phenomenon such as restitution and need to be adjusted to generate results that qualitatively resemble VF. As such, while our P4 model quantitatively matches some metrics of VF measured in rabbit hearts, further validation would increase confidence, and our P4 model is *not* appropriate for phenomena in which restitution, intracellular calcium and other factors play a role. Since these phenomena are probably important in VF initiation, we believe that our results should be interpreted only in regard to VF maintenance.

Overall, our results, when interpreted with previous simulations with the same heart meshes (Bishop and Plank, 2012; Pathmanathan and Gray, 2015) provide concrete new insights. First, one should *not* assume that simulations of reentry in the whole heart with cell models that are stable or meander in 2-D will result in monomorphic of polymorphic tachycardia because fibrillation-like behavior may occur. We have shown that both fine structure and macroscopic heterogeneities including fibers act to destabilize reentrant waves in simulations incorporating bi-ventricular geometries. We argue that our simulations of VF using parameter set P4 using the Bishop rabbit heart mesh may represent the best correspondence to experimental results in the isolated rabbit heart to date.

## Author Contributions

SG: performed simulations, post processing, analyzing data, figure preparation, video preparation, performed review of literature, and wrote the initial draft of paper. PP: wrote the code for detecting filaments, analyzed data, and edits on all aspects of paper. MB: provided the rabbit meshes and provided feedback on paper. RG: conception of the experiment, analyzing data, and edits on all aspects of paper.

### Conflict of Interest Statement

The authors declare that the research was conducted in the absence of any commercial or financial relationships that could be construed as a potential conflict of interest.
