# Supplementary material for: Effect of Heart Structure on Ventricular Fibrillation in the Rabbit: A Simulation Study
Source: Front Physiol. 2019 May 15;10:564. doi: 10.3389/fphys.2019.00564 (PMC6536150; doi:10.3389/fphys.2019.00564)
Supplement: Supplementary file 17 [file Data_Sheet_2.docx]

**SUPPLEMENTARY MATERIAL**

1. Figures
   1. Snapshots of activity
   2. Sensitivity of filament dynamics to system configuration for anisotropic simulations with structure
2. Movie legends
3. **Figures**
   1. **Snapshots of activity**

**
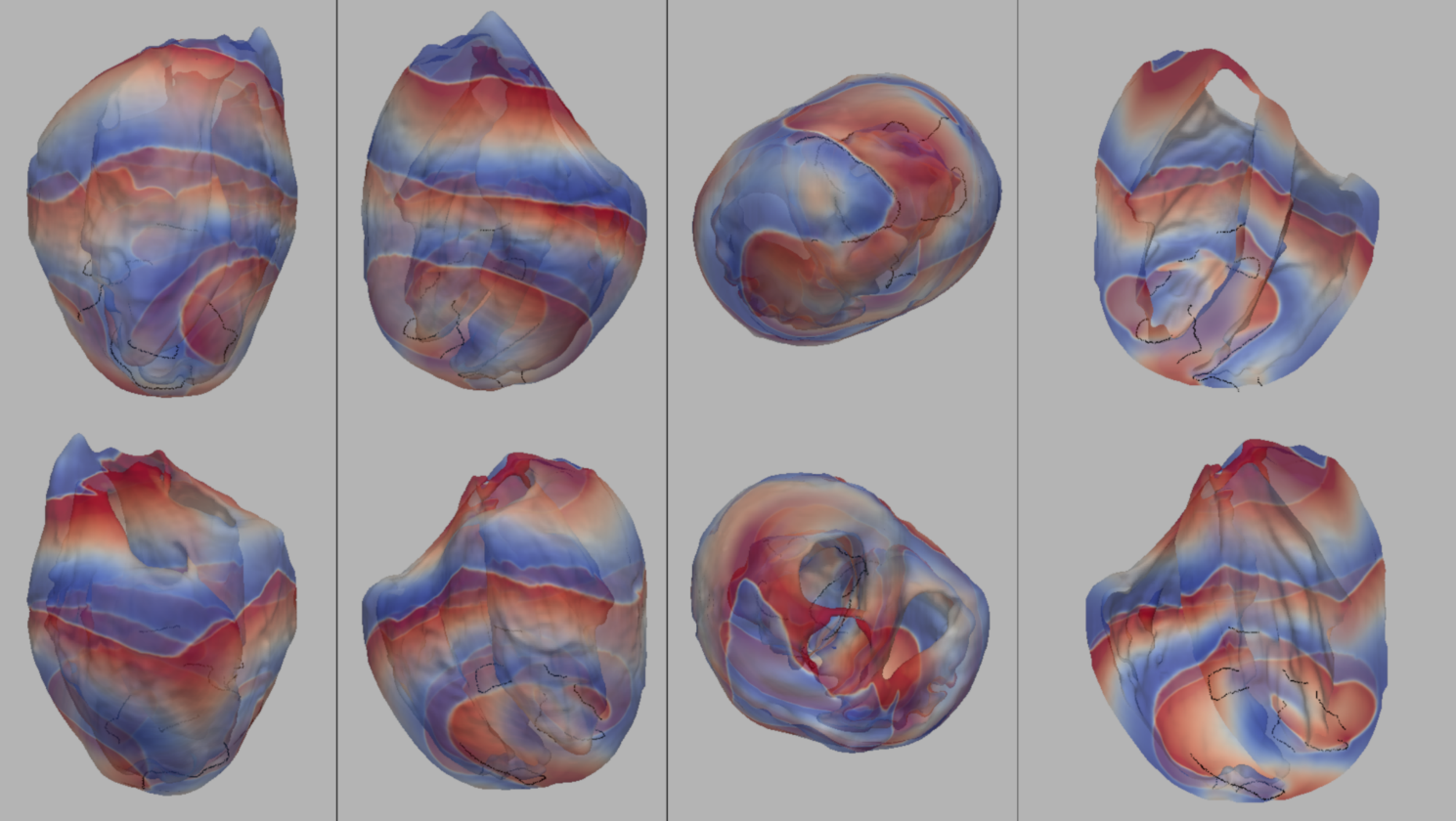
**

**SFigure1.** Multiple views of simulated fibrillation for P1_A_nS at 320 ms after initiation. Transmembrane potential is represented with a blue-red color map such that blue corresponds to -83 mV and red to +20 mV. Filaments are shown as thin black curves.


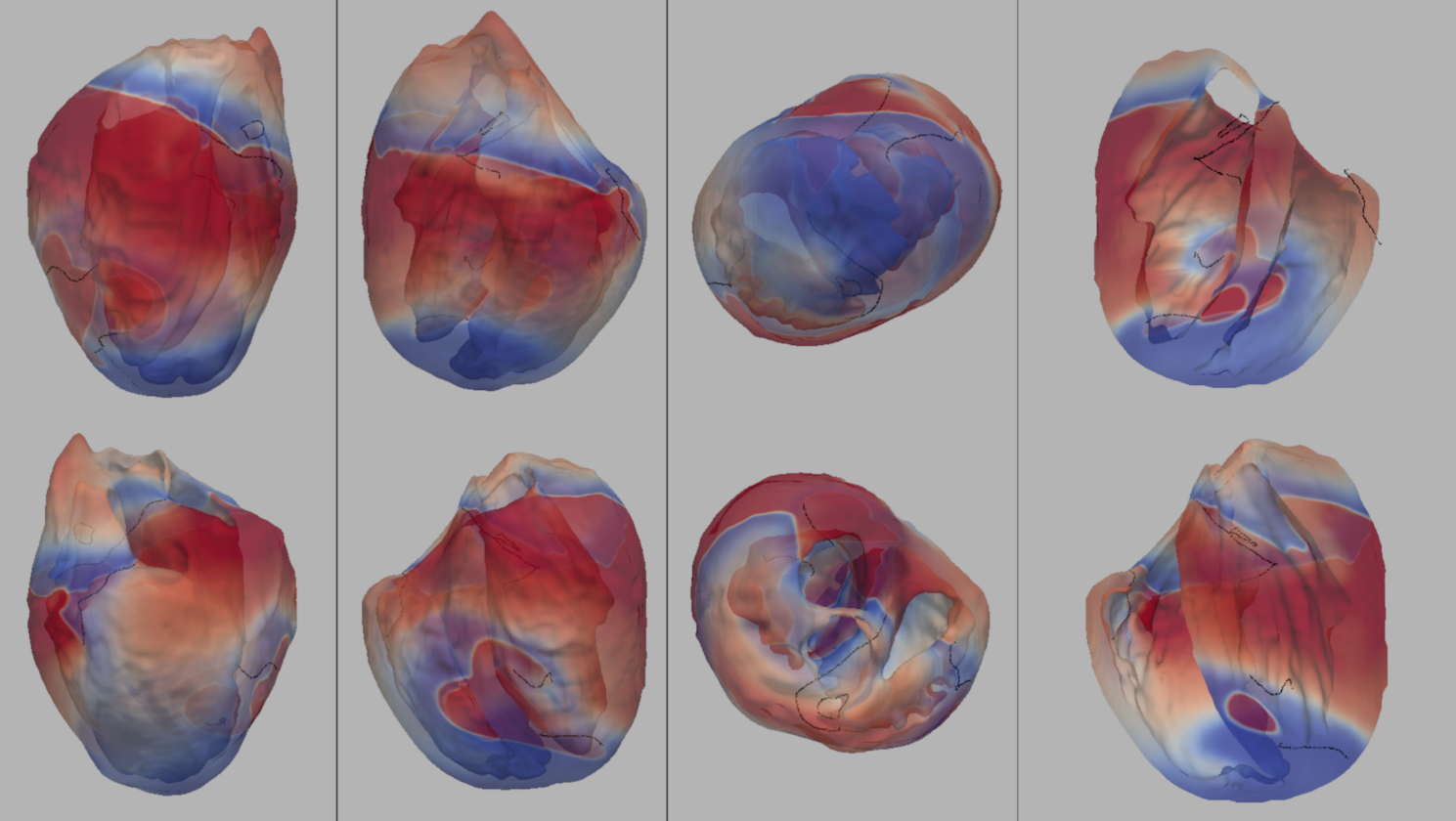


**SFigure2.** Multiple views of simulated fibrillation for P3_A_nS at 320 ms after initiation. Transmembrane potential is represented with a blue-red color map such that blue corresponds to -83 mV and red to +20 mV. Filaments are shown as thin black curves.


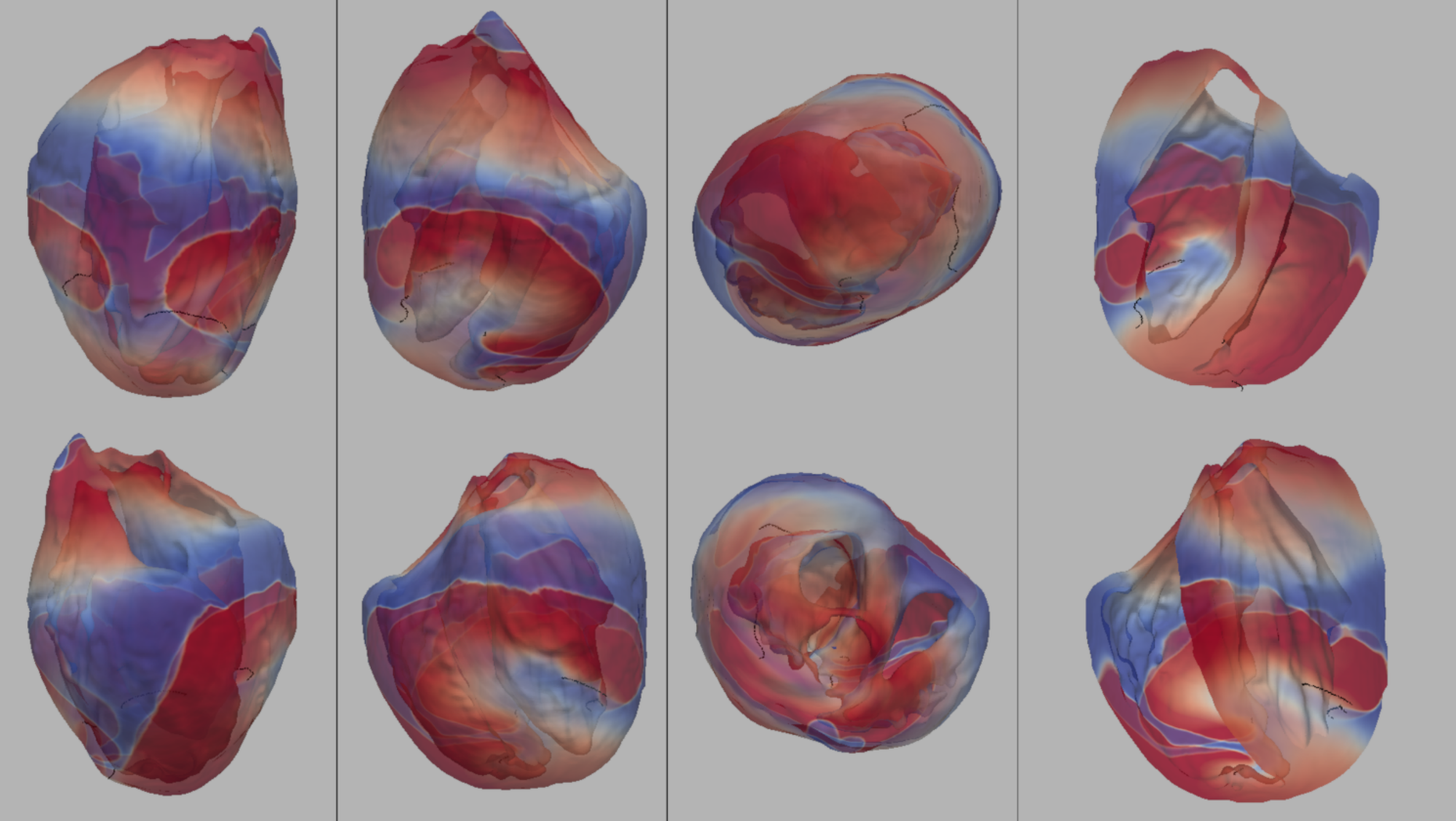


**SFigure3.** Multiple views of simulated fibrillation for P4_A_nS at 320 ms after initiation. Transmembrane potential is represented with a blue-red color map such that blue corresponds to -83 mV and red to +20 mV. Filaments are shown as thin black curves.


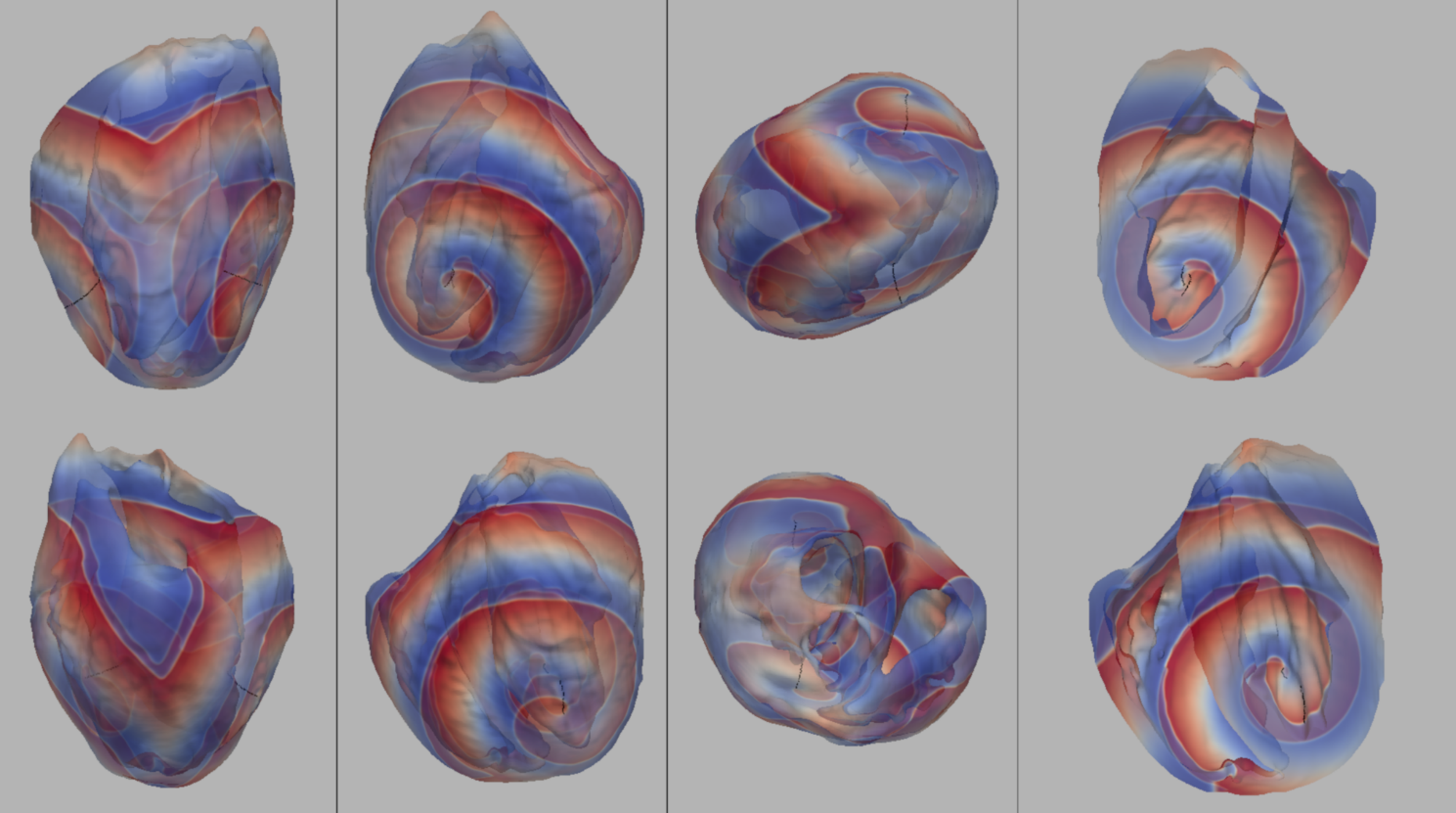


**SFigure4.** Multiple views of simulated fibrillation for P1_I_nS at 320 ms after initiation. Transmembrane potential is represented with a blue-red color map such that blue corresponds to -83 mV and red to +20 mV. Filaments are shown as thin black curves.


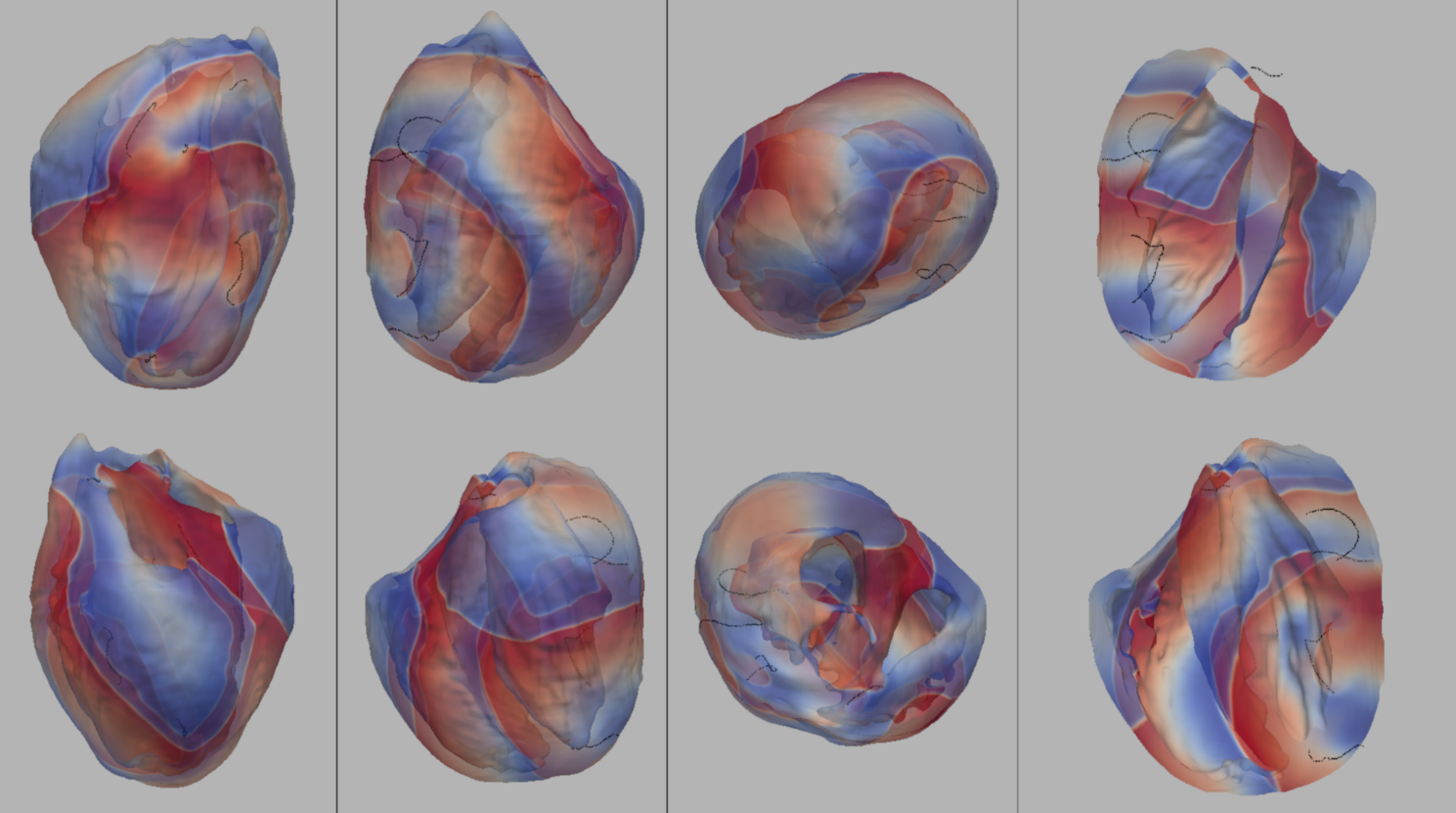


**SFigure5.** Multiple views of simulated fibrillation for P2_I_nS at 320 ms after initiation. Transmembrane potential is represented with a blue-red color map such that blue corresponds to -83 mV and red to +20 mV. Filaments are shown as thin black curves.


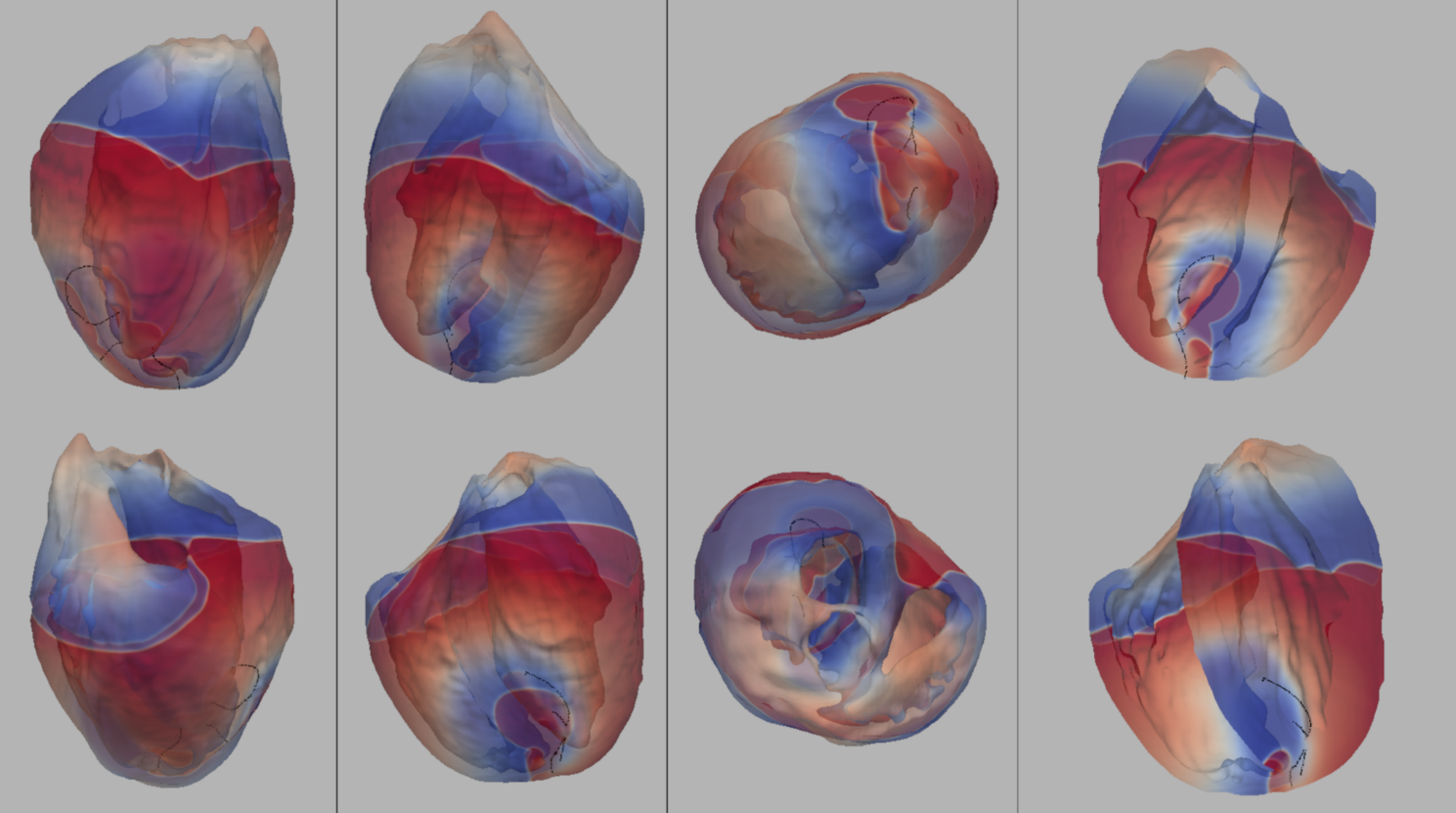


**SFigure6.** Multiple views of simulated fibrillation for P3_I_nS at 320 ms after initiation. Transmembrane potential is represented with a blue-red color map such that blue corresponds to -83 mV and red to +20 mV. Filaments are shown as thin black curves.


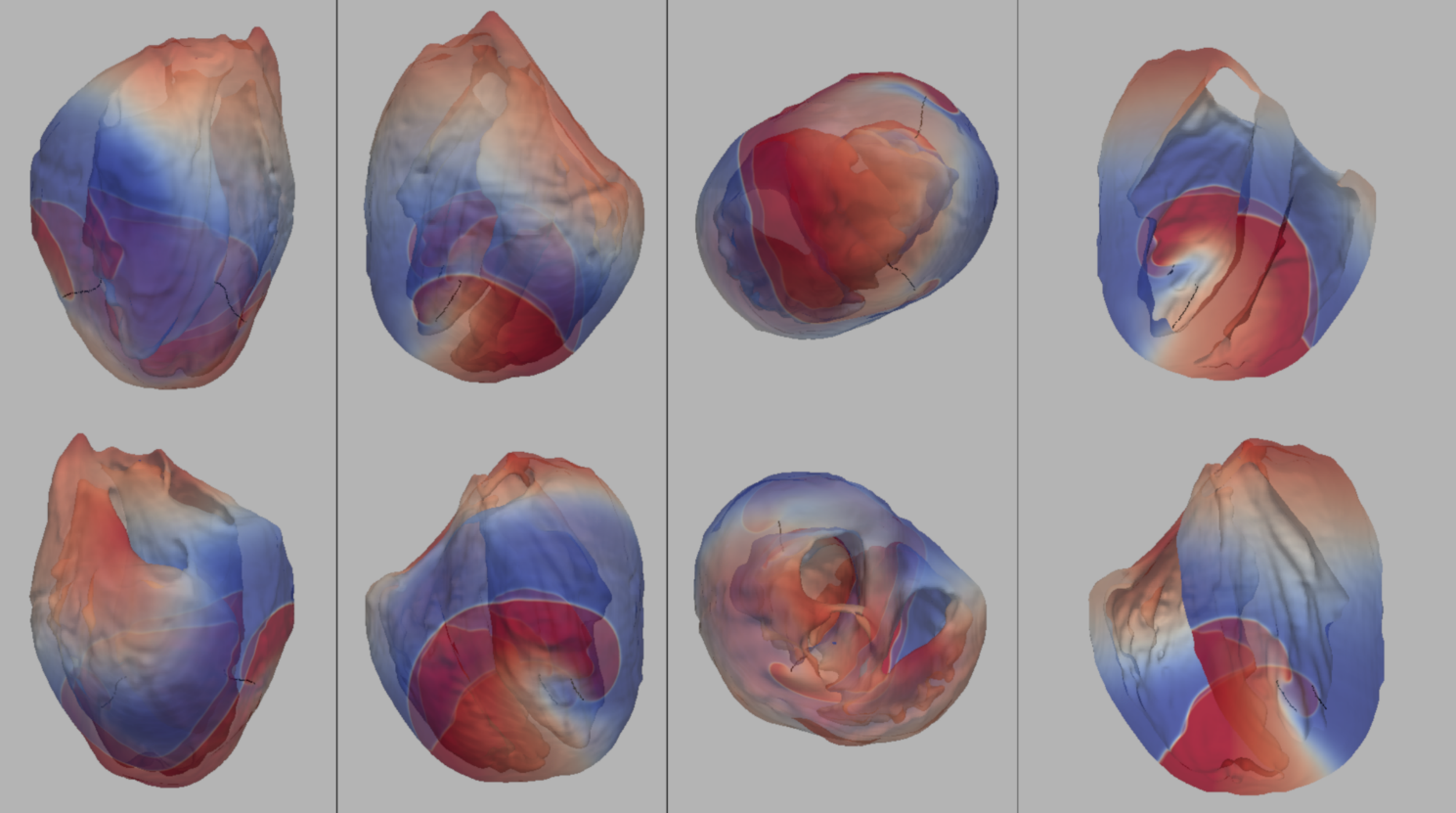


**SFigure7.** Multiple views of simulated fibrillation for P4_I_nS at 320 ms after initiation. Transmembrane potential is represented with a blue-red color map such that blue corresponds to -83 mV and red to +20 mV. Filaments are shown as thin black curves.


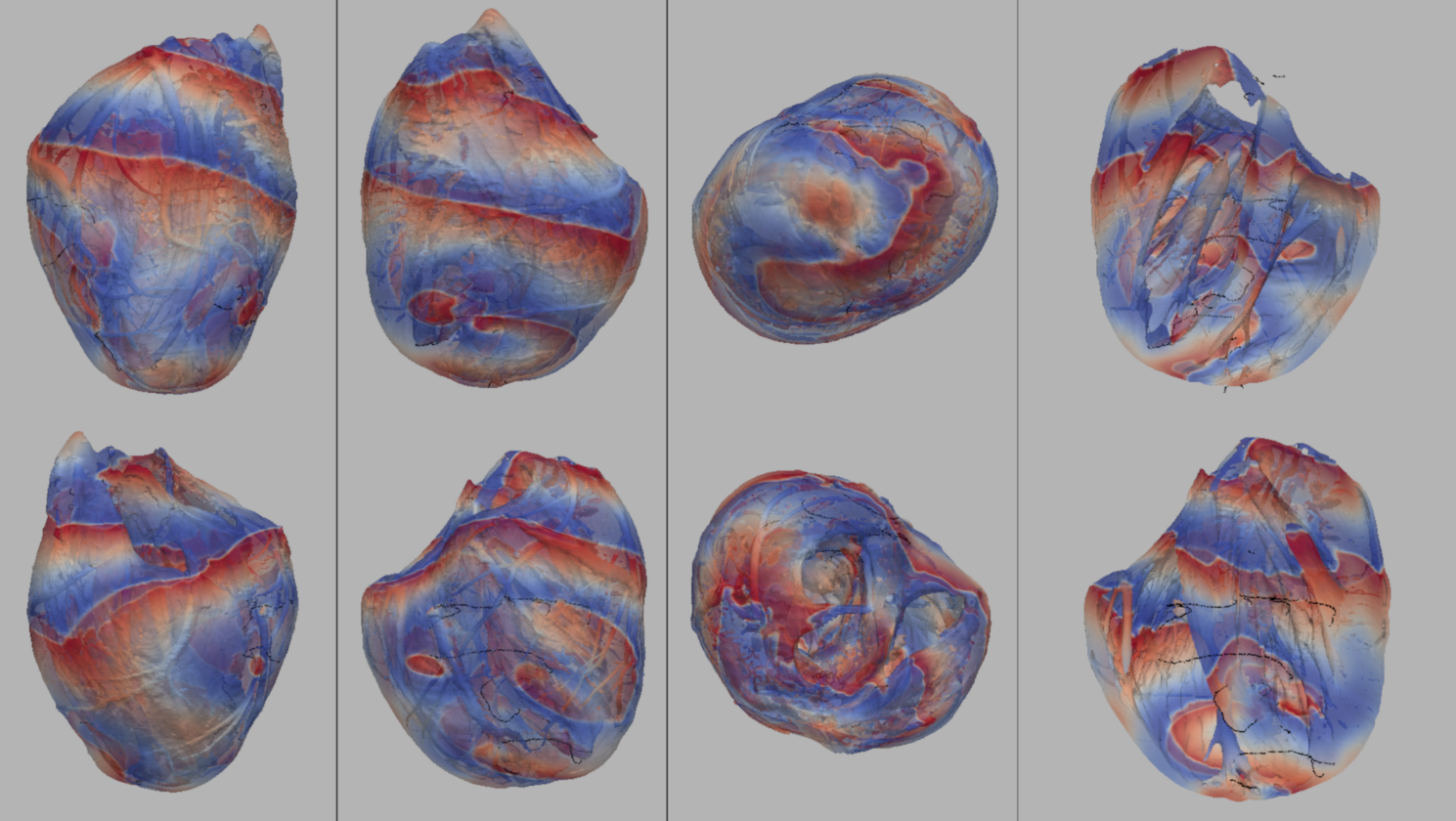


**SFigure8.** Multiple views of simulated fibrillation for P1_A_wS at 320 ms after initiation. Transmembrane potential is represented with a blue-red color map such that blue corresponds to -83 mV and red to +20 mV. Filaments are shown as thin black curves.


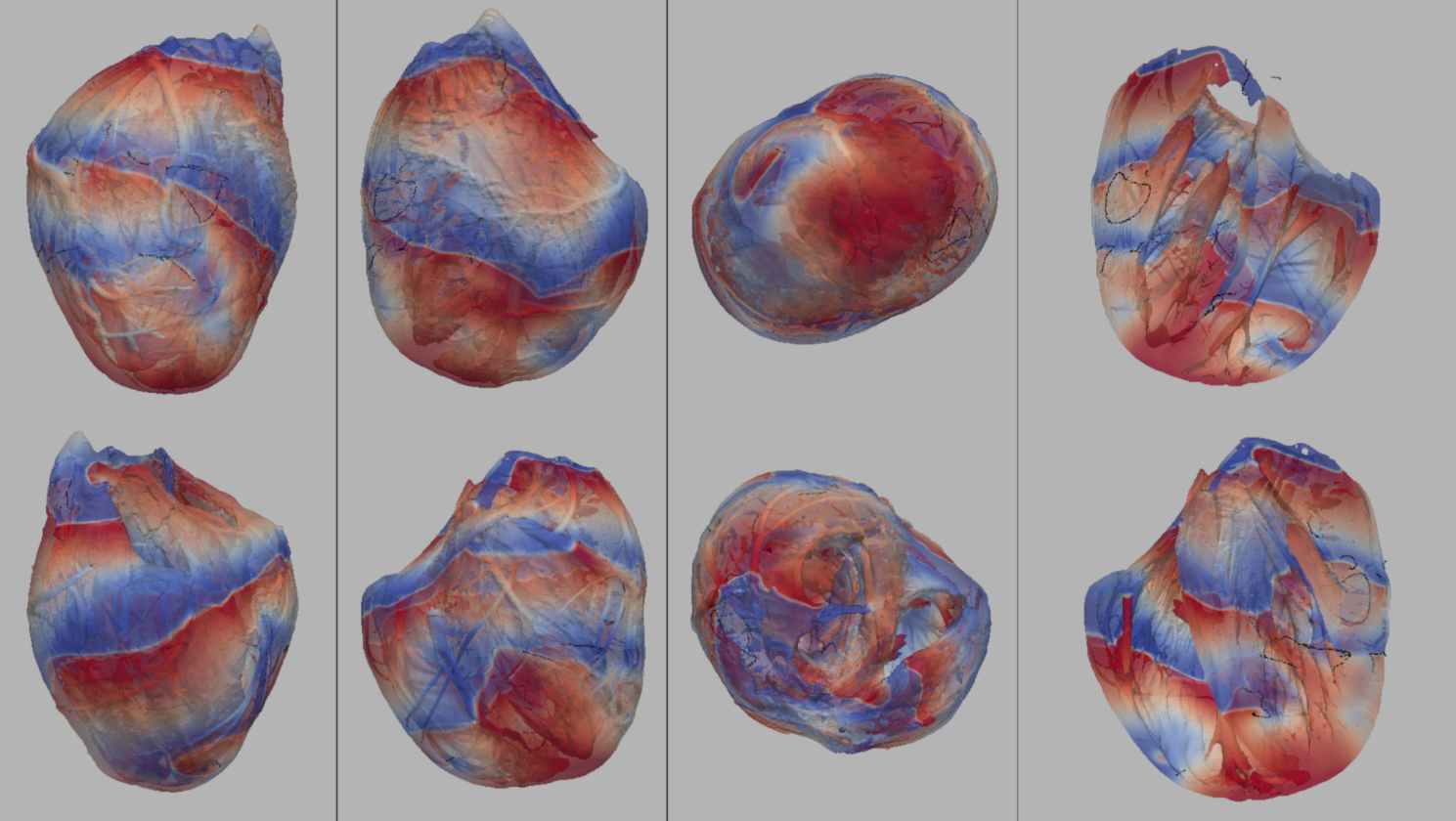


**SFigure9.** Multiple views of simulated fibrillation for P2_A_wS at 320 ms after initiation. Transmembrane potential is represented with a blue-red color map such that blue corresponds to -83 mV and red to +20 mV. Filaments are shown as thin black curves.


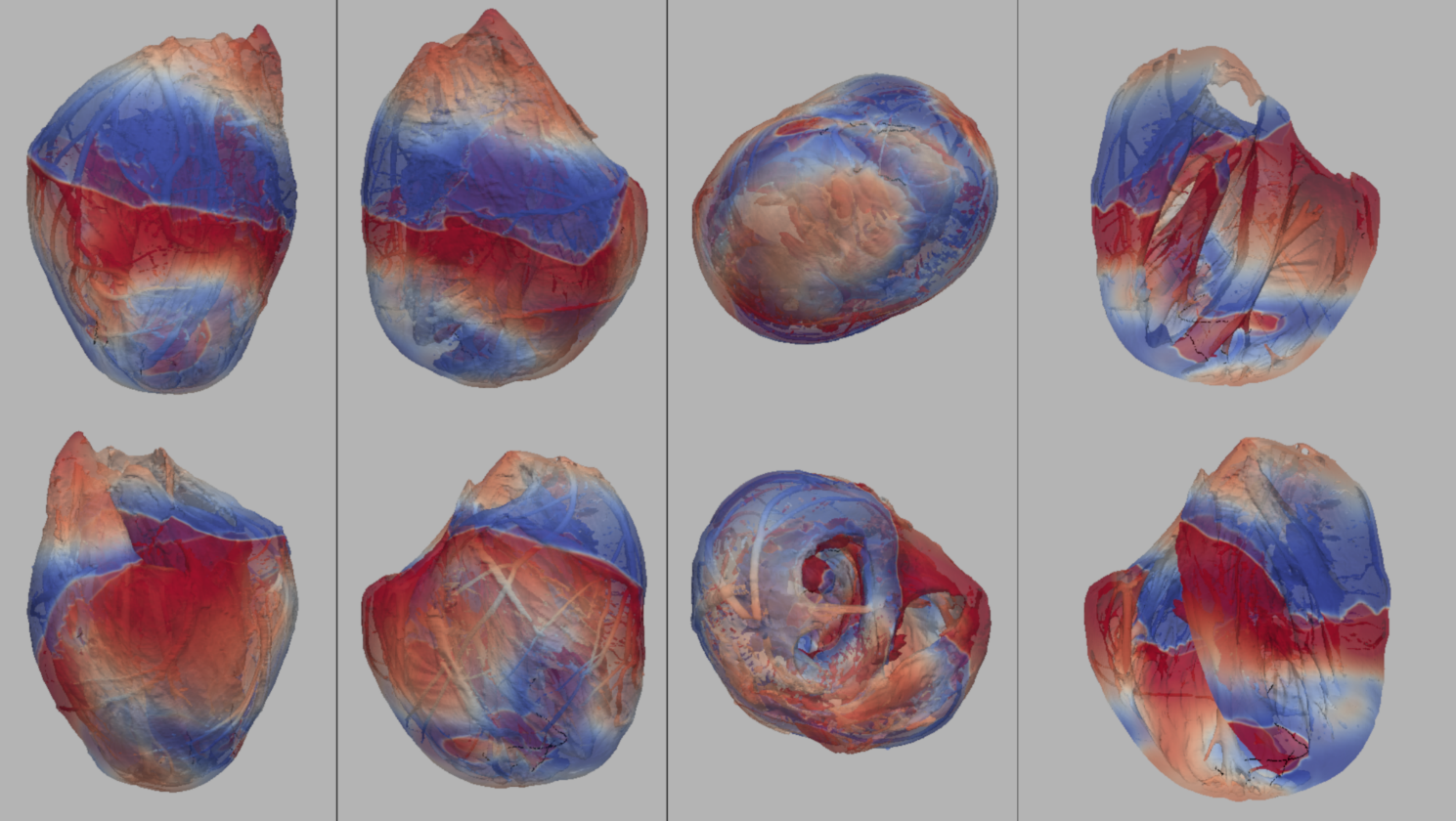


**SFigure10.** Multiple views of simulated fibrillation for P3_A_wS at 320 ms after initiation. Transmembrane potential is represented with a blue-red color map such that blue corresponds to -83 mV and red to +20 mV. Filaments are shown as thin black curves.


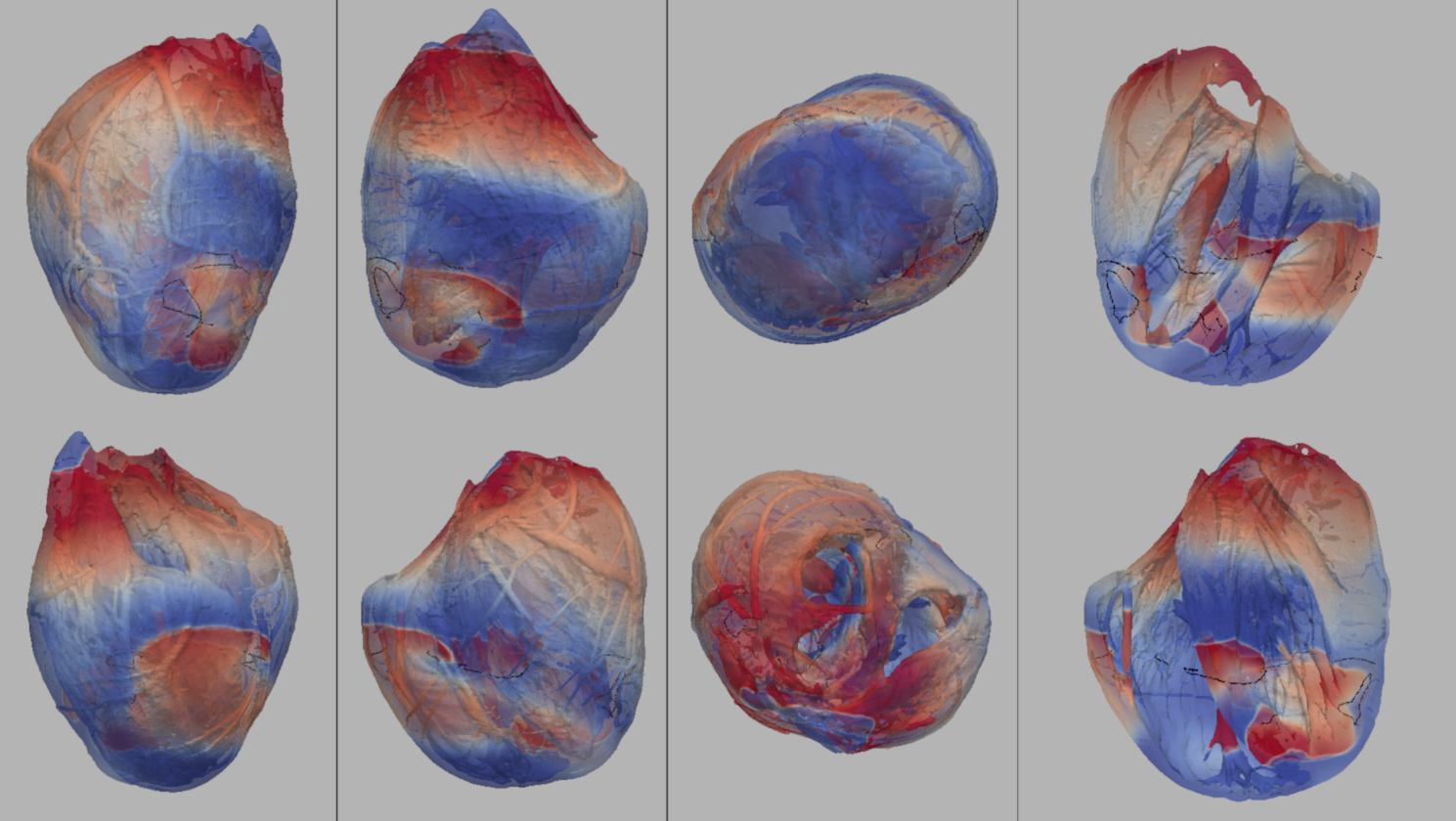


**SFigure11.** Multiple views of simulated fibrillation for P4_A_wS at 320 ms after initiation. Transmembrane potential is represented with a blue-red color map such that blue corresponds to -83 mV and red to +20 mV. Filaments are shown as thin black curves.


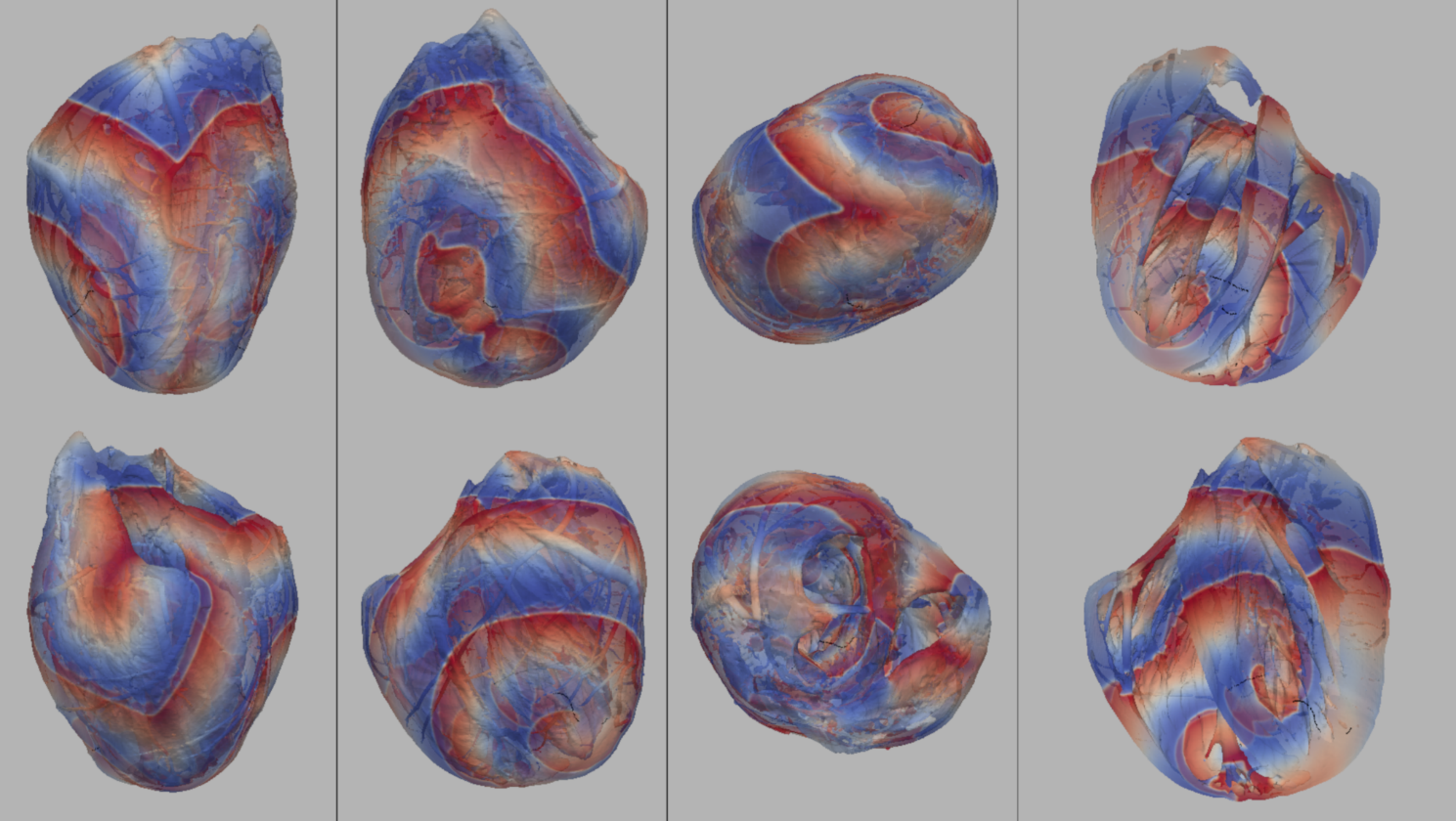


**SFigure12.** Multiple views of simulated fibrillation for P1_I_wS at 320 ms after initiation. Transmembrane potential is represented with a blue-red color map such that blue corresponds to -83 mV and red to +20 mV. Filaments are shown as thin black curves.


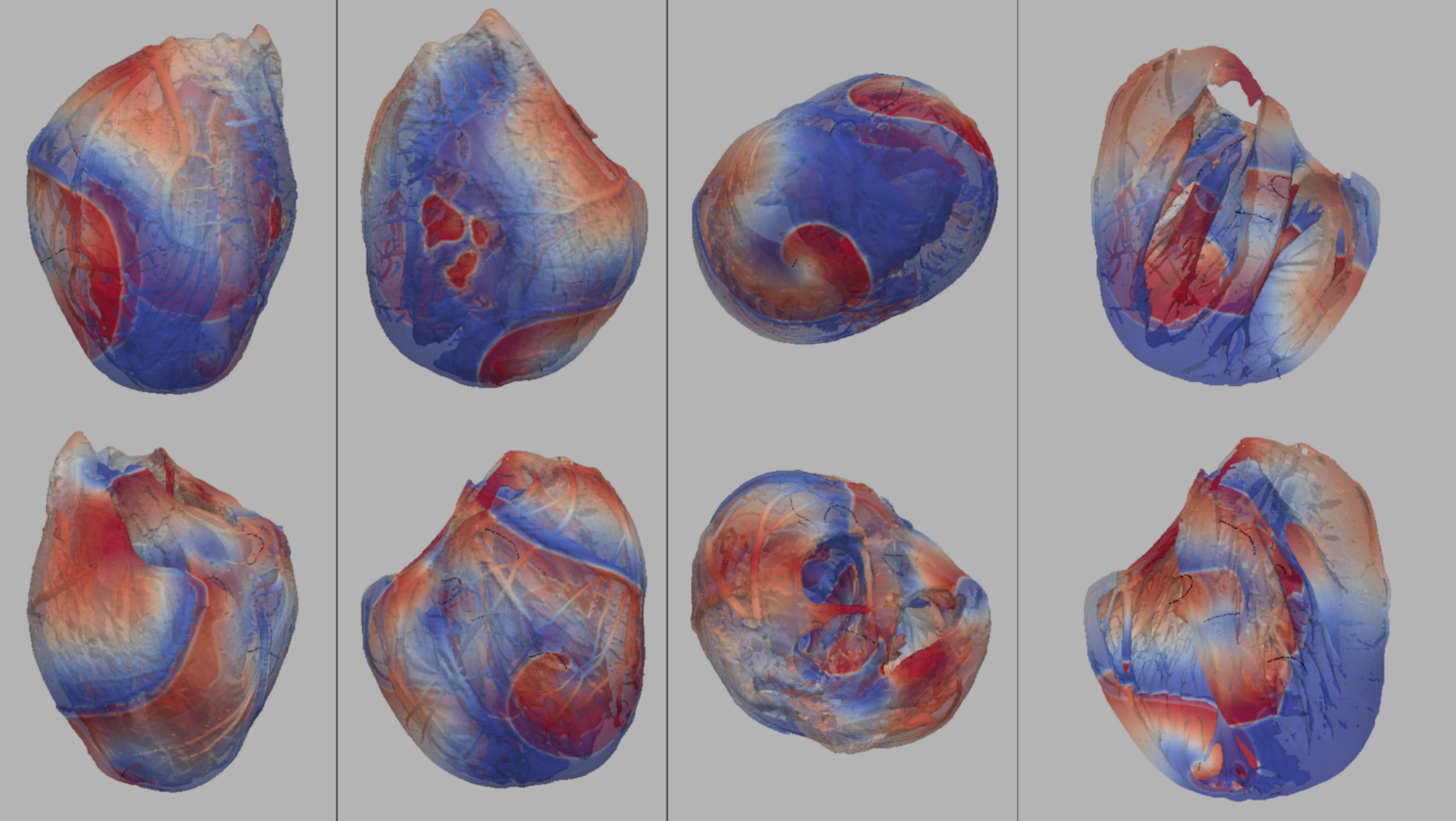


**SFigure13.** Multiple views of simulated fibrillation for P2_I_wS at 320 ms after initiation. Transmembrane potential is represented with a blue-red color map such that blue corresponds to -83 mV and red to +20 mV. Filaments are shown as thin black curves.


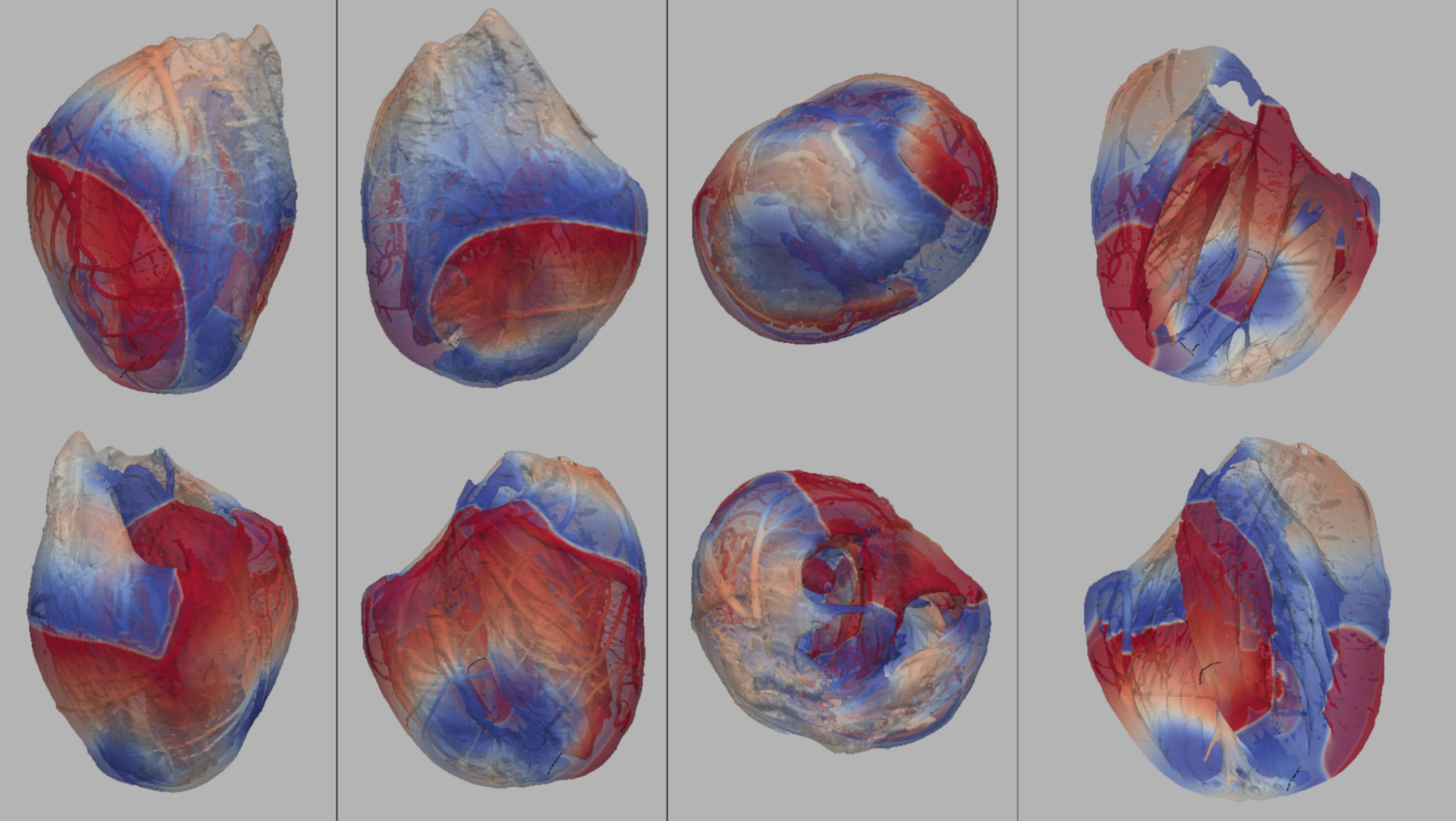


**SFigure14.** Multiple views of simulated fibrillation for P3_I_wS at 320 ms after initiation. Transmembrane potential is represented with a blue-red color map such that blue corresponds to -83 mV and red to +20 mV. Filaments are shown as thin black curves.


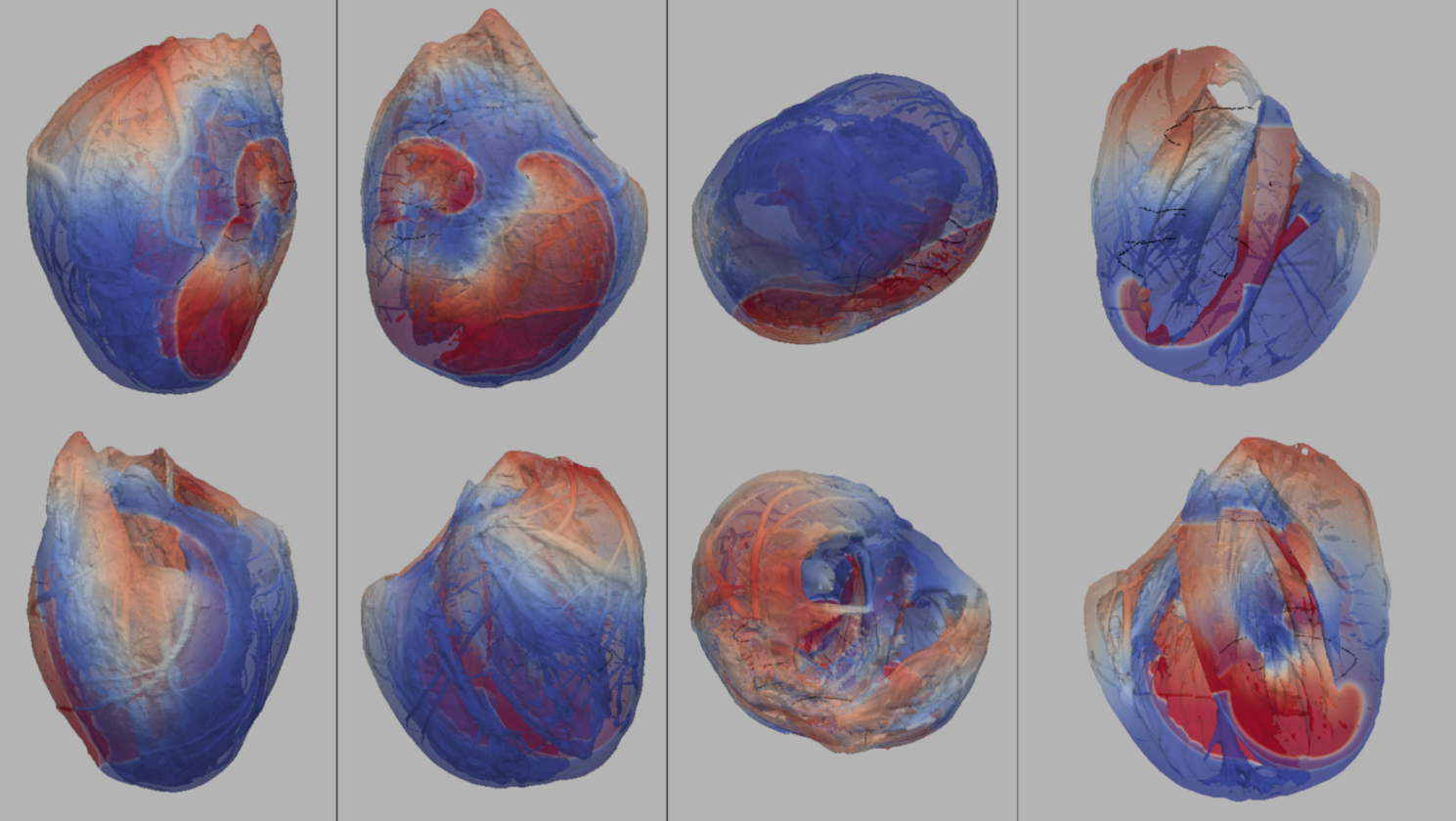


**SFigure15.** Multiple views of simulated fibrillation for P4_I_wS at 320 ms after initiation. Transmembrane potential is represented with a blue-red color map such that blue corresponds to -83 mV and red to +20 mV. Filaments are shown as thin black curves.

- 1. **Sensitivity of filament dynamics to system configuration for anisotropic simulations with structure**


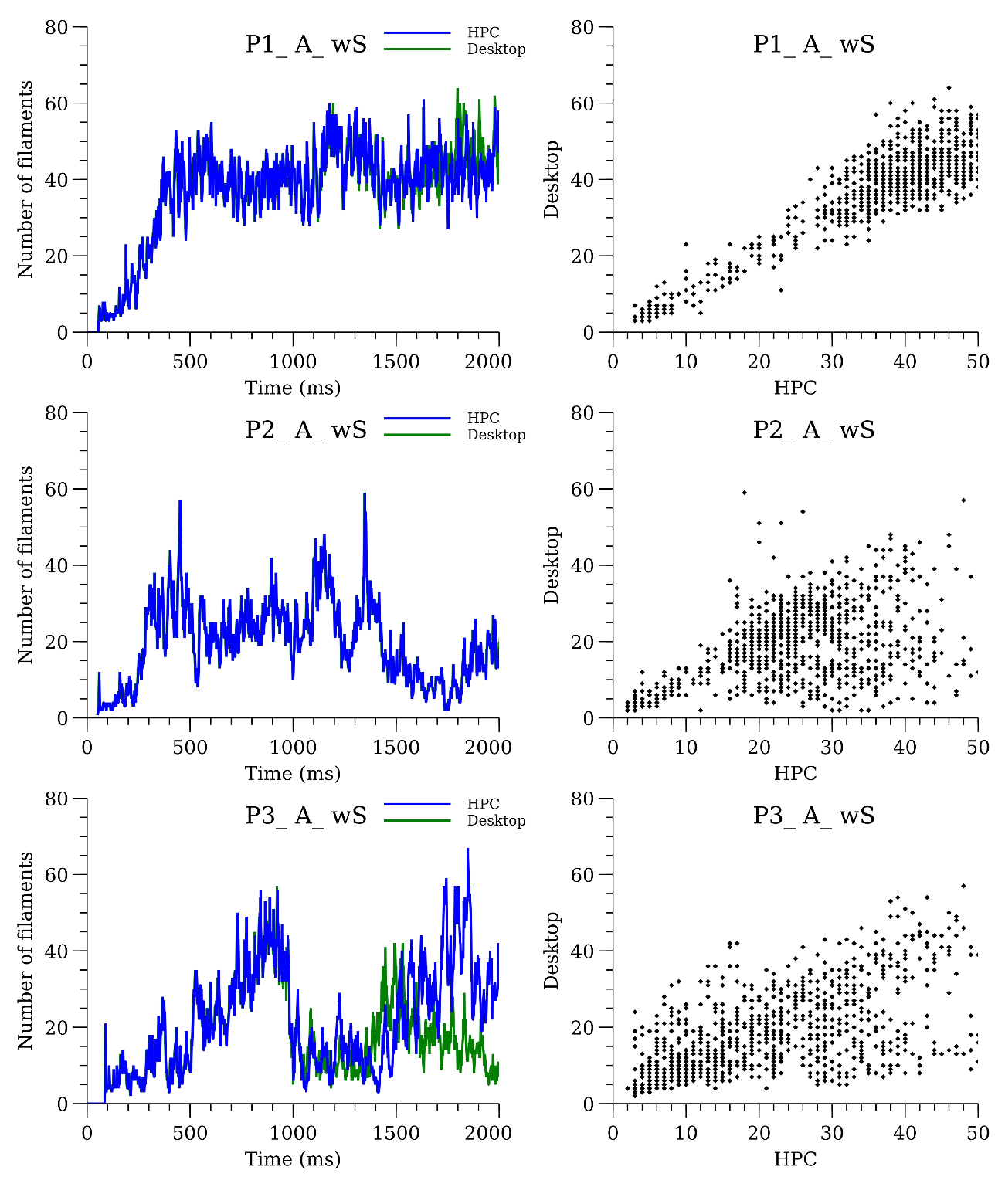


**SFigure16.** Sensitivity of filament dynamics to system configuration for anisotropic simulations with structure. The plots on the left-hand side show the number of filaments over time for desktop (green line) and HPC (blue line) configurations. The plots on the right show the divergence between the two configurations.

1. **Movie legends**

**SMovie1.** Multiple views of simulated fibrillation for P1_I_nS for the first 2 seconds of activity. Transmembrane potential is represented with a blue-red color map such that blue corresponds to -83 mV and red to +20 mV. Filaments are shown as thin black curves.

**SMovie2.** Multiple views of simulated fibrillation for P2_I_nS for the first 2 seconds of activity. Transmembrane potential is represented with a blue-red color map such that blue corresponds to -83 mV and red to +20 mV. Filaments are shown as thin black curves.

**SMovie3.** Multiple views of simulated fibrillation for P3_I_nS for the first 2 seconds of activity. Transmembrane potential is represented with a blue-red color map such that blue corresponds to -83 mV and red to +20 mV. Filaments are shown as thin black curves.

**SMovie4.** Multiple views of simulated fibrillation for P4_I_nS for the first 2 seconds of activity. Transmembrane potential is represented with a blue-red color map such that blue corresponds to -83 mV and red to +20 mV. Filaments are shown as thin black curves.

**SMovie5.** Multiple views of simulated fibrillation for P1_I_wS for the first 2 seconds of activity. Transmembrane potential is represented with a blue-red color map such that blue corresponds to -83 mV and red to +20 mV. Filaments are shown as thin black curves.

**SMovie6.** Multiple views of simulated fibrillation for P2_I_wS for the first 2 seconds of activity. Transmembrane potential is represented with a blue-red color map such that blue corresponds to -83 mV and red to +20 mV. Filaments are shown as thin black curves.

**SMovie7.** Multiple views of simulated fibrillation for P3_I_wS for the first 2 seconds of activity. Transmembrane potential is represented with a blue-red color map such that blue corresponds to -83 mV and red to +20 mV. Filaments are shown as thin black curves.

**SMovie8.** Multiple views of simulated fibrillation for P4_I_wS for the first 2 seconds of activity. Transmembrane potential is represented with a blue-red color map such that blue corresponds to -83 mV and red to +20 mV. Filaments are shown as thin black curves.

**SMovie9.** Multiple views of simulated fibrillation for P1_A_nS for the first 2 seconds of activity. Transmembrane potential is represented with a blue-red color map such that blue corresponds to -83 mV and red to +20 mV. Filaments are shown as thin black curves.

**SMovie10.** Multiple views of simulated fibrillation for P2_A_nS for the first 2 seconds of activity. Transmembrane potential is represented with a blue-red color map such that blue corresponds to -83 mV and red to +20 mV. Filaments are shown as thin black curves.

**SMovie11.** Multiple views of simulated fibrillation for P3_A_nS for the first 2 seconds of activity. Transmembrane potential is represented with a blue-red color map such that blue corresponds to -83 mV and red to +20 mV. Filaments are shown as thin black curves.

**SMovie12.** Multiple views of simulated fibrillation for P4_A_nS for the first 2 seconds of activity. Transmembrane potential is represented with a blue-red color map such that blue corresponds to -83 mV and red to +20 mV. Filaments are shown as thin black curves.

**SMovie13.** Multiple views of simulated fibrillation for P1_A_wS for the first 2 seconds of activity. Transmembrane potential is represented with a blue-red color map such that blue corresponds to -83 mV and red to +20 mV. Filaments are shown as thin black curves.

**SMovie14.** Multiple views of simulated fibrillation for P2_A_wS for the first 2 seconds of activity. Transmembrane potential is represented with a blue-red color map such that blue corresponds to -83 mV and red to +20 mV. Filaments are shown as thin black curves.

**SMovie15.** Multiple views of simulated fibrillation for P3_A_wS for the first 2 seconds of activity. Transmembrane potential is represented with a blue-red color map such that blue corresponds to -83 mV and red to +20 mV. Filaments are shown as thin black curves.

**SMovie16.** Multiple views of simulated fibrillation for P4_A_wS for the first 2 seconds of activity. Transmembrane potential is represented with a blue-red color map such that blue corresponds to -83 mV and red to +20 mV. Filaments are shown as thin black curves.
